# Cr^2+^ solid solution in UO_2_ evidenced by advanced spectroscopy

**DOI:** 10.1038/s42004-022-00784-3

**Published:** 2022-12-01

**Authors:** Hannah Smith, Luke T. Townsend, Ritesh Mohun, Théo Cordara, Martin C. Stennett, J. Frederick W. Mosselmans, Kristina Kvashnina, Claire L. Corkhill

**Affiliations:** 1 NucleUS Immobilisation Science Laboratory, Department of Materials Science and Engineering, The University of Sheffield, Sheffield, UK; 2 Diamond Light Source, Harwell Science and Innovation Campus, Didcot, UK; 3 Helmholtz-Zentrum Dresden-Rossendorf (HZDR), Institute of Resource Ecology, PO Box 510119, 01314 Dresden, Germany; 4 The Rossendorf Beamline at ESRF – The European Synchrotron, Grenoble, France

**Keywords:** Materials science, Energy science and technology, Techniques and instrumentation, Nuclear energy, Ceramics

## Abstract

Advanced Cr-doped UO_2_ fuels are essential for driving safe and efficient generation of nuclear energy. Although widely deployed, little is known about their fundamental chemistry, which is a critical gap for development of new fuel materials and radioactive waste management strategies. Utilising an original approach, we directly evidence the chemistry of Cr^(3+)^_2_O_3_–doped U^(4+)^O_2_. Advanced high-flux, high-spectral purity X-ray absorption spectroscopy (XAS), corroborated by diffraction, Raman spectroscopy and high energy resolved fluorescence detection-XAS, is used to establish that Cr^2+^ directly substitutes for U^4+^, accompanied by U^5+^ and oxygen vacancy charge compensation. Extension of the analysis to heat-treated simulant nuclear fuel reveals a mixed Cr^2+/3+^ oxidation state, with Cr in more than one physical form, explaining the substantial discrepancies that exist in the literature. Successful demonstration of this analytical advance, and the scientific underpinning it provides, opens opportunities for an expansion in the range of dopants utilised in advanced UO_2_ fuels.

## Abstract (web summary)

Chromia is an important additive used in uranium dioxide fuel fabrication, but its incorporation mechanism is still not fully understood. Here, the authors use X-ray absorption spectroscopy, including both near edge and extended fine structure regions, to resolve the local structure and valence state of chromium, as divalent, in uranium dioxide.

## Introduction

Chromium (Cr) is an important additive used in uranium dioxide (UO_2_) fuel fabrication. The doping of Cr, added as Cr^(3+)^_2_O_3_, into UO_2_ fuels leads to enhanced grain growth, realising beneficial properties of higher fission gas retention and reduced swelling during reactor operations. During the subsequent long-term storage and disposal of such fuels post-fission, the dissolution behaviour in aqueous media, a two-stage mechanism dependent upon the oxidation of U^(4+)^O_2_ to more soluble U^(6+)^O_2_^2+^, will be integral to safety over millions of years^1,2^. Since doping of the UO_2_ lattice with aliovalent species will alter the valence state of U and, consequently, the local defect structure of UO_2_, e.g. forming U^(4+/5+)^O_2 + x_, an understanding of the influence of Cr on the UO_2_ lattice is required to support safety assessments for disposal. While the role of Cr on practical aspects such as fuel performance^3–7^ and grain growth mechanisms^8–13^ is generally understood, agreement on the mechanism of Cr incorporation into the UO_2_ structure and its consequent impacts on U chemistry has proven elusive^14–21^. One reason for this is the prior focus on materials that have been sintered to mimic nuclear fuel production, on which a number of factors, such as pressure, temperature and atmosphere will influence the O diffusion kinetics in the material and, therefore, the final valence, distribution and coordination of U and Cr in the UO_2_ structure. Since these sintered systems are highly complex, the lattice structures between studies tend to be incomparable due to the varying sintering conditions used.

A number of possible incorporation mechanisms and associated speciation of Cr within UO_2_ have been proposed, implicating Cr^3+^ or Cr^2+^, and in one case, Cr^1+^. A substitution mechanism is proposed, evidenced by a reduction in the lattice parameter of UO_2_ upon doping, attributed to substitution of smaller Cr^2+/3+^ (ionic radii of 0.73 Å and 0.62 Å, respectively^22^) on the larger U^4+^ (ionic radius 1.00 Å^22^) site. This mechanism requires charge compensation *via* positive defect formation; for example, U^5+^ species and/or oxygen vacancy (O_v_) defects are expected to develop, causing further lattice distortion. Comparison of Cr K-edge X-ray Absorption Spectra (XAS), in particular, the X-ray Absorption Near Edge (XANES) spectral region of sintered Cr-doped UO_2_ pellets with a range of Cr standards, has previously provided tentative evidence for the Cr^3+^ valence state^20,23^. More recent ab initio atomistic simulations, based on qualitative assessment of XANES data, argue the plausibility of Cr^2+^ speciation^21^, with substitution onto the U^4+^ site and concurrent formation of one O_v_, determined to be the most thermodynamically favourable configuration. Alternately, in density functional theory and empirical potential descriptions, where the valence state of Cr was not restrained to the trivalent state, modelling of defect concentrations in Cr-doped UO_2_ at high temperature has predicted Cr^1+^ interstitial defects would be the dominant species^8^.

To gain definitive experimental evaluation of the Cr incorporation mechanism, quantitative information relating to the lattice interatomic distances and near-neighbour coordination, through the use of Extended X-ray Absorption Fine Structure (EXAFS) region of XAS data is required. The only reported EXAFS fitting of Cr-doped UO_2,_ carried out on commercially available and chemically complex sintered nuclear fuel pellets, gave evidence that Cr is incorporated as Cr^3+^ in 6-fold coordination^23^. The feasibility of Cr^2+^ valence and the presence of undissolved Cr^(2+)^O phases in the pellet was discussed, but not verified. This preliminary work reported a significant change in Cr-O bond length (2.02 ± 0.02 Å) when compared to the U-O distance (2.36 ± 0.02 Å) in UO_2_, which was attributed to disruption in both the cation-cation and cation-anion lattices due to the presence of Cr.

In the current study, a high-flux, high spectral-purity beamline was utilised to fully resolve the XAS Cr K-edge, including both XANES and EXAFS regions, thus making it possible to fully resolve the local structure and valence state of Cr in UO_2_. Additionally, high-energy resolution fluorescence detection (HERFD) XANES data were acquired at the U M_4_-edge to evidence the oxidation state of U^24,25^, complemented by Raman spectroscopy analysis of the Cr-doped UO_2_ oxygen defect chemistry. Importantly, our study avoids the ambiguity induced in the interpretation of sintered UO_2_ by focusing on simple heat treated (i.e., calcined, not sintered) materials. We thus develop an experimentally-evidenced, fundamental understanding of the mechanism of Cr_2_O_3_ incorporation within UO_2_ oxide powder. This understanding is further applied to Cr-doped UO_2_ sintered in a reducing atmosphere, where the unavoidable complexity introduced into the system, and the associated challenge in assigning incorporation mechanisms, is demonstrated.

## Results and discussion

### Cr-speciation in UO_2_ calcined powder

**Fig. 1:**
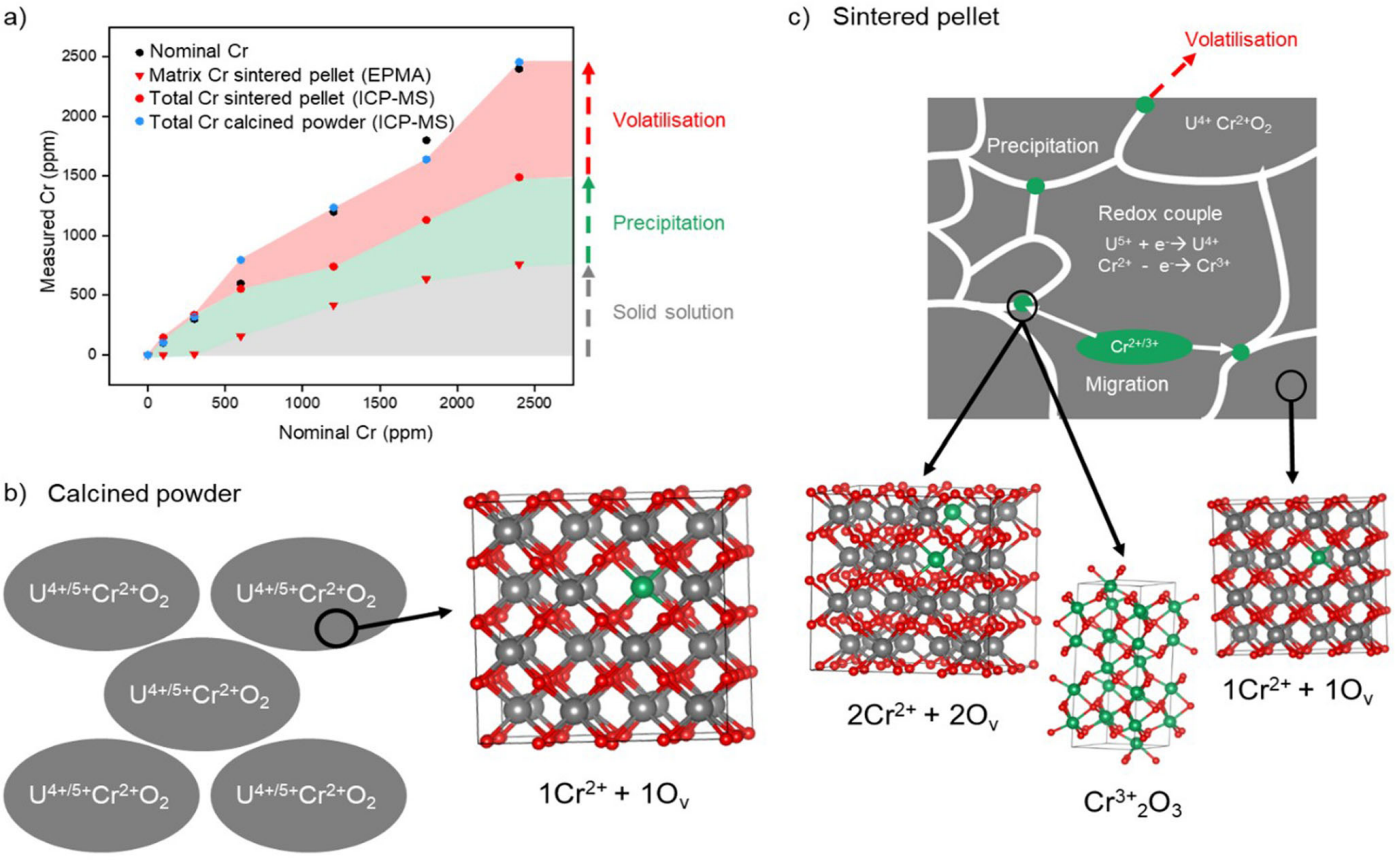
Cr content and incorporation in UO_2_. **a** The measured Cr content of Cr-doped UO_2_ calcined powder and sintered pellets. Total Cr content measured by ICP-MS following acid digest and matrix content of sintered samples measured by EPMA, excluding grain boundary precipitates; it highlights the proportion of Cr that is incorporated in the UO_2_ matrix in solid solution, incorporated as precipitates and Cr that has been lost from the system due to volatilisation; **b** schematic representation and associated crystal structure of particles of calcined Cr-doped UO_2_^21^ and; **c** schematic representation and crystal structures of the sintered matrix of Cr-doped UO_2_^21,26^, highlighting distribution of Cr in precipitates (green) at grain boundaries (white). In the crystal structures, grey atoms represent U, green atoms represent Cr and red represent O.

Confirmation of successful Cr-doping of UO_2_ powder upon calcination was given by ICP-MS analysis. All oxide powders were shown to contain the intended concentration of Cr when fully digested (in the range 300–2400 ppm) (Fig. 1a).

**Fig. 2:**
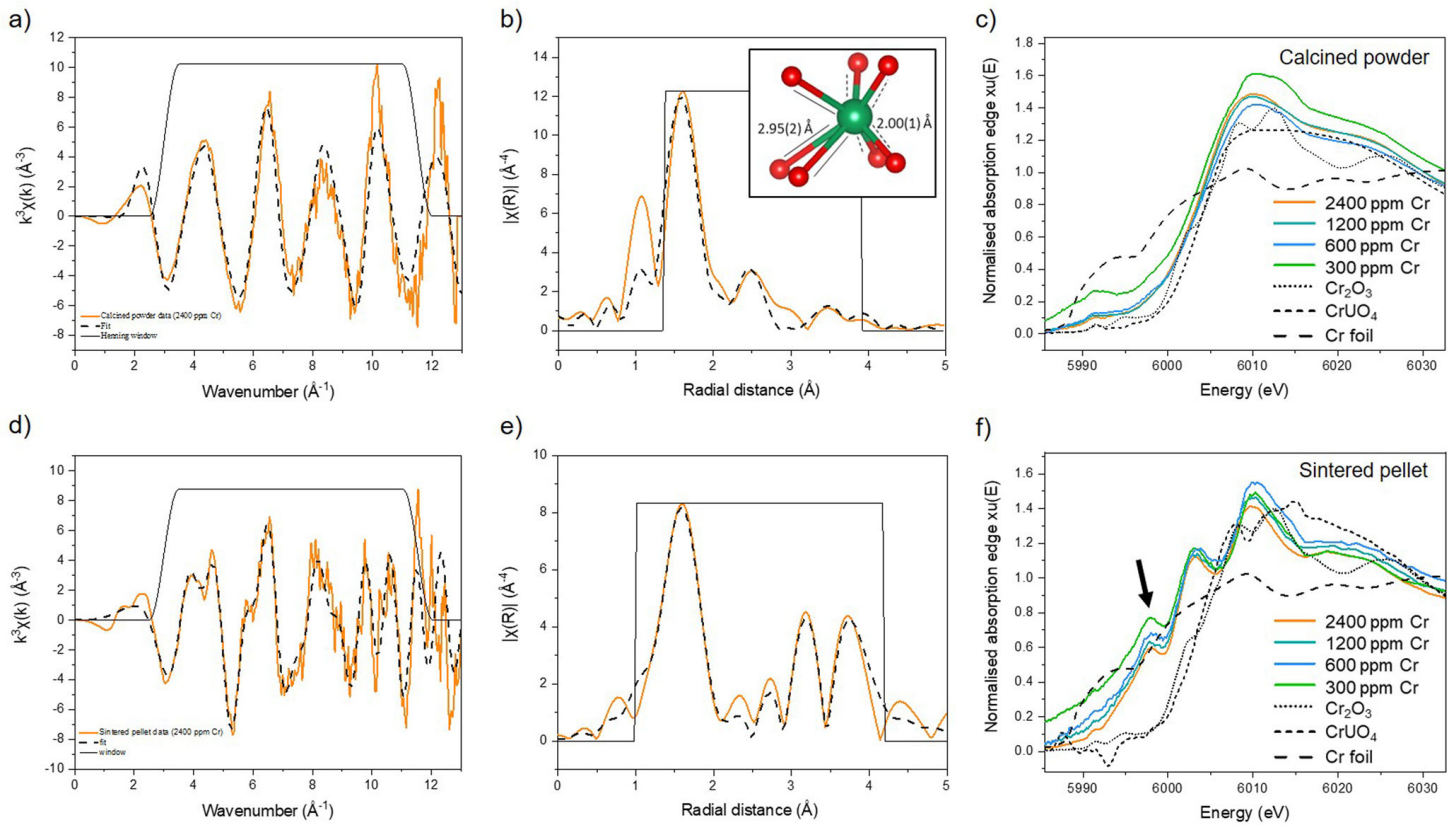
Crystal structure and local coordination analysis of Cr in Cr-doped UO_2_ determined by EXAFS and XANES analysis. k^3^-weighted Cr K-edge spectra, EXAFS model fits and Cr K-edge XANES spectra of Cr-doped UO_2_ in comparison to known standards (Cr_2_O_3_, CrUO_4_ and Cr^0^ foil), respectively, for (**a**–**c**) calcined powders; and (**d**–**f**) sintered pellets. For (**a**, **b**, **d**, **e**) orange lines are data and black dashed lines are the fits.

The approach to Cr K-edge EXAFS fitting considered a range of different structural models (a selection of which are shown in Supplementary Fig. 1 and Supplementary Table 1), including simple fits that only contained oxygen backscatterers, as well as fits based upon models from the DFT work of Sun et al.^21^. The best fit for the calcined material was obtained using a structure derived from ab initio atomistic models of Cr-doped UO_2_, in which Cr^2+^ substitutes onto the U^4+^ cation site *via* the formation of O_v_ for charge balance, denoted by (1Cr^2+^ + 1O_v_)^21^ (Fig. 1b). The EXAFS model (Fig. 2a, b, Table 1) indicates that the first O nearest neighbour environment is split, with 4 O atoms at a distance of 2.00 ± 0.01 Å, and 3 O atoms at 2.95 ± 0.02 Å, giving a total coordination of 7. Since this is less than the expected coordination of 8, it implies that one O_v_ is situated in the Cr coordination environment. Confirmation of Cr^2+^ speciation is given by the bond valence sum (BVS)^27^ of 2.05. The second shell Cr–U environment was successfully fitted by the model, with 4 U atoms at a scattering distance of 3.58 ± 0.04 Å and 2 U atoms at 3.85 ± 0.06 Å. These latter Cr–U distances are in good agreement with U–U distances of UO_2_ (3.83 Å^23^) while the presence of shorter Cr–U distances can be attributed to distortion of U atoms adjacent to the O_v_. Detailed fit results are displayed in Table 1. This data supports the hypothesis of Sun et al.^21^ who found that the most thermodynamically favourable structural arrangement of Cr in UO_2_, through ab initio calculations, is a pair of associated Cr^2+^ and O_v_, i.e. 1Cr^2+^ + 1O_v_. Given the excellent statistics of the fit (R-factor and corroborating BVS), it is unlikely that any other Cr species exist in the calcined sample, with its bulk speciation being accurately described by the 1Cr^2+^ + 1O_v_ model.

**Table 1:** Fitting parameters and results for 2400 ppm Cr-doped UO_2_ calcined oxide powder and sintered pellet samples.

	UO_2_ + 2400 ppm Cr (Calcined oxide powder)	UO_2_ + 2400 ppm Cr (Sintered pellet)
*S*_0_^2^	0.90	0.90
∆*E*_o_	−0.80(2)	−6.0(15)
*N* (Cr-O1)	4	4.5
*R*_eff_ (Cr-O1)	2.10*	2.10*^
R(Cr-O1)	2.00(1)	2.00(1)
σ^2^ (O1)	0.0010(6)	0.005(1)
α (%)	100.0	100.0
*N* (Cr-O2)	3	1.5
*R*_eff_ (Cr-O2)	2.94*	2.17′
*R*(Cr-O2)	2.95(2)	2.44(2)
σ^2^ (O2)	0.004(3)	0.002(2)
α (%)	99.6	100.0
*N* (Cr-U1)	4	3
*R*_eff_ (Cr-U1)	3.49*	3.49*^
*R*(Cr-U1)	3.58(4)	3.40(7)
σ^2^ (U1)	0.017(6)	0.014(8)
α (%)	85.7	100.0
*N* (Cr-U2)	2	1
*R*_eff_ (Cr-U2)	3.81*	3.40*
*R*(Cr-U2)	3.85(6)	3.34(5)
σ^2^ (U2)	0.010(7)	0.004(3)
α (%)	79.1	100.0
*N* (Cr-U3)	–	2
*R*_eff_ (Cr-U3)	–	3.81*^
*R*(Cr-U3)	–	3.91(2)
σ^2^ (U3)	–	0.001(1)
α (%)	–	100.0
R-factor	0.019	0.013
Bond valence sum (O1)	1.94	2.14
Bond valence sum (O2)	0.11	0.22
Bond valence sum (total)	2.05	2.35

*S*_0_^2^ is the amplitude reduction factor, ∆*E*_0_ the shift from Cr K-edge position (5989 eV), *N* the degeneracy, *R*_eff_ (Å) the reference bond length, *R* (Å) the fitted bond length, σ^2^ the Debye–Waller factor, and α F-test factor.

Structures used to inform path fitting *1Cr+1O_v_, ’2Cr+2O_v,_ ^Cr_2_O_3_.

**Fig. 3:**
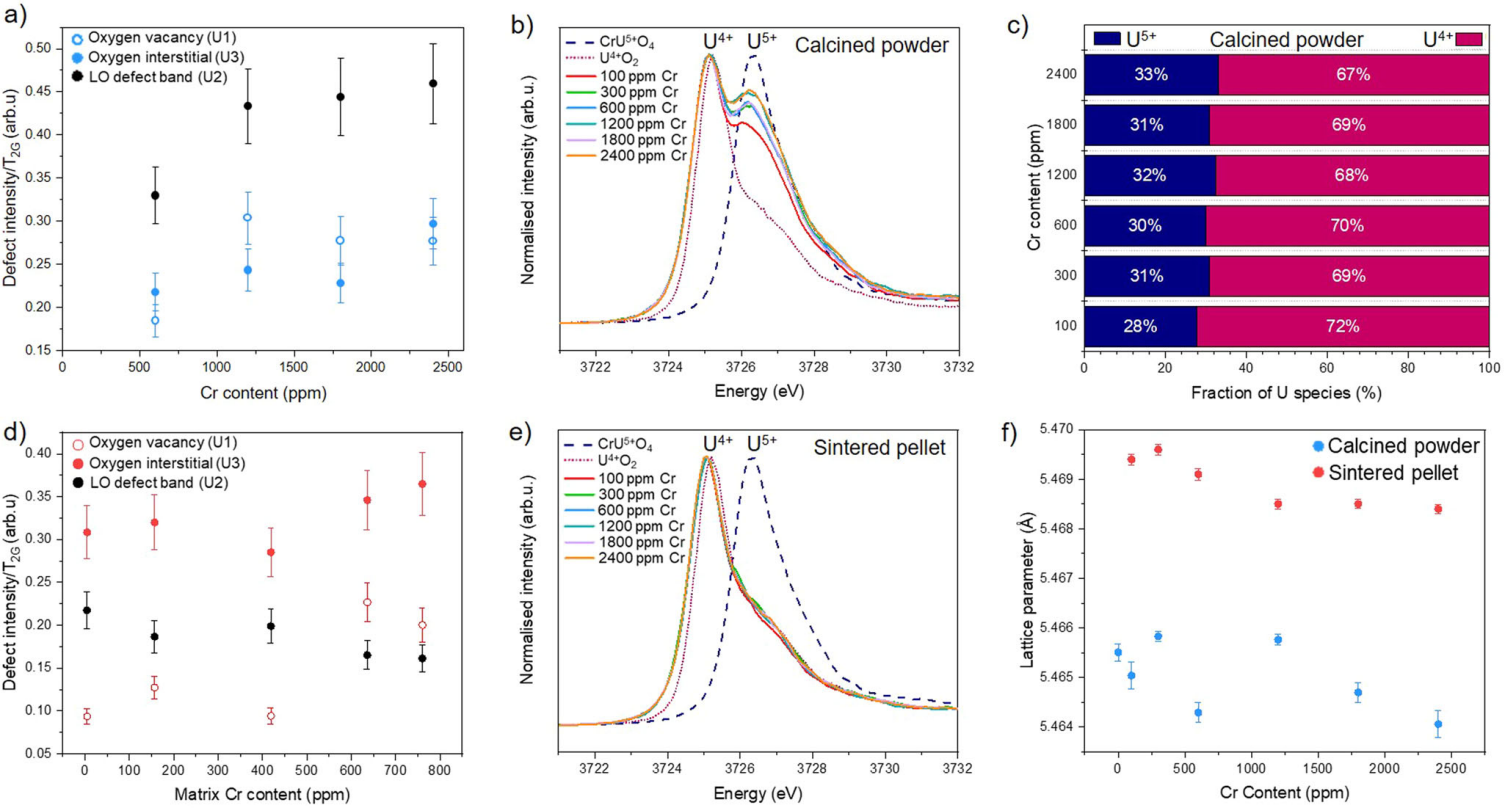
Raman Spectroscopy, U M_4_-edge HERFD XANES and p-XRD analysis of Cr-doped UO_2_ materials. **a** Oxygen defect content realised by deconvolution of Raman spectra of calcined material (errors derived from the standard deviation of 10 measurements); **b** U M_4_-edge HERFD XANES of calcined material. **c** Quantification of the U^4+/5+^ ratio in calcined powder determined from fitting of U M_4_-edge HERFD XANES data in b. **d** Oxygen defect content of sintered material realised by deconvolution of Raman spectra (errors derived from the standard deviation of 10 measurements). **e** U M_4_-edge HERFD XANES of sintered material; and (**f**) lattice parameter values for calcined powder and sintered material, as determined by p-XRD (errors derived from the lattice parameter refinement).

The substitution mechanism via vacancy formation is further supported by an increase in the O_v_ (U1) defects observed with increasing Cr-dopant concentration, according to Raman measurements (Fig. 3a). HERFD XANES data acquired at the U M_4_-edge (Fig. 3b, Supplementary Fig. 2) showed, through principal component analysis of the data series, that only two components were required to accurately reconstruct each of the samples in the series. These were attributed to U^5+^ and U^4+^ oxidation states using the standards CrU^5+^O_4_ and U^4+^O_2_. Whilst the higher energy component matches well with the U^5+^ standard, U^4+^ shows some differences when compared to the UO_2_ standard. This is likely due to the distortion around the U^4+^ coordination environment upon doping of UO_2_ with Cr, possibly due to vacancy formation. Iterative target transformation factor analysis was used to indicate the approximate fraction of U^4+^ and U^5+^ in the Cr-doped oxide powders (Fig. 3c), with results indicating ~ 30% U^5+^ (and therefore ~70% U^4+^) for all samples. This corroborates the hypothesis that charge balance for Cr^2+^ within Cr-doped UO_2_ is provided by both O_v_ and U^5+^ defect formation, with O_v_ potentially playing a more prominent role. The final stoichiometry is, therefore, $({({U}_{1-x}^{4+}{U}_{x}^{5+})}_{1-y}C{r}_{y}^{2+}){O}_{2-\frac{y}{2}}$.

Analysis of the UO_2_ T_2g_ Raman band at 445 cm^−1^, and other peaks relevant to defect structures in the matrix (Fig. 3a and Supplementary Fig. 3a, b), revealed a slight increase in the oxygen interstitial (O_i_) (U3) concentration with increasing Cr-dopant concentration. Given that the concentrations of Cr in the calcined material exceed the generally agreed solubility limit of Cr in UO_2_ (reported to be in the range of 700–1200 ppm^3,6,9,11,15^, it could be assumed that excess Cr may be present as positive interstitial defects^8^. If this were the case, negative O_i_ defects would be required as a charge balance mechanism. Since the O_i_ defect concentration (Fig. 3a) broadly increases with increasing Cr content, this seems to be a plausible explanation. However, it is also possible that such defects arise from slight oxidation of the sample during analysis. Given that the EXAFS model did not require the presence of Cr interstitials to improve the fit, the latter explanation may be more reasonable.

### Cr-speciation in sintered UO_2_ pellets

The complexity induced during sintering (analogous to nuclear fuel synthesis) was investigated using calcined Cr-doped UO_2_ powders pressed into pellets and heat-treated (sintered) at 1700 °C under a reducing 5%H_2_:95%N_2_ atmosphere. Loss of Cr, expected due to high temperature volatilisation^28,29^, is observed through a reduction in the Cr concentration when compared with calcined powder (Fig. 1a). In contrast to the homogenous Cr incorporation in calcined powders, significant heterogeneity in the distribution of Cr in the sintered pellets was observed (Supplementary Fig. 4). This is exemplified by the precipitation of Cr-containing particles within grain boundaries, which has previously been observed for Cr-doped UO_2_ sintered under a range of conditions^6,9,11,12,15,20^.

Retention of a portion of Cr within the UO_2_ matrix upon sintering is evidenced by a reduction in the lattice parameter with increasing Cr-dopant matrix concentration, as measured by powder X-ray diffraction (p-XRD, Fig. 3f). The absolute values of lattice parameter between the sintered and calcined oxide material are different, with the latter being lower than the former by ~0.004 Å. This is most likely due to the loss of some Cr^2+^ in the lattice due to volatilisation (Fig. 1a). Alternatively, the lattice parameter decrease could result from the reduction of Cr^2+^ to Cr^0^ (ionic radius 2.00 Å) in the reducing sintering atmosphere or, conversely, possible oxidation of pellets after sintering. Both alternative explanations are excluded on the basis of experimental data obtained: the absence of U^5+^ and U^6+^ in the HERFD-XANES data for the sintered material (Fig. 3e) and a lower O_i_ concentration than in the calcined materials (Fig. 3d), precludes enhanced oxidation. Moreover, analysis of the XAS data effectively excludes the presence of Cr^0^ within the EXAFS model (see below).

In accordance with the highly reducing sintering conditions, the white line position (*E*_0_; when taken as the peak of the first derivative; Supplementary Data Table 2) of the Cr K-edge (~6000 eV, Fig. 2f) is shifted to lower energy than that of the Cr^3+^ standards (6006.0 eV) and the calcined Cr^2+^UO_2_ material (6003.2 eV), and towards that of the Cr^0^ standard (5989 eV). The presence of Cr^2+^, rather than Cr^0^, in the sintered material is confirmed by the occurrence of the pre-edge feature at 5995 eV, which originates from the forbidden 1 s → 4 s transition of Cr^2+^ ^30^. This transition becomes allowed for Cr^2+^ only when multiple Cr^2+^ environments are present, which induces s to p orbital mixing. The lower the symmetry of the Cr^2+^ environments, the more that such hybridisation can occur; consequently, it is proposed that multiple, low-symmetry Cr^2+^ environments exist for the sintered material. That this transition is not observed as strongly in the equivalent calcined Cr^2+^-containing UO_2_ XANES spectra (Fig. 2c), is likely to be a consequence of the single Cr^2+^ coordination and high symmetry environment of those materials.

In comparison with the calcined material, fitting of the EXAFS data acquired from the sintered materials was challenging. As with the calcined material, several models were first explored; however, all produced unsatisfactory fits, both quantitatively and qualitatively. The best fit was achieved by utilising the 1Cr^(2+)^+1O_v_ structure^21^ in addition to a second Cr^2+^ environment of 2Cr^(2+)^+ 2O_v_ and also a Cr^3+^ environment, of Cr^(3+)^_2_O_3_ (Fig. 2e and Table 1; structures shown in Fig. 1c). The mixed Cr^2+^/Cr^3+^ oxidation state is confirmed by the resultant BVS, which gives an average Cr oxidation state of 2.35. The EXAFS fit was used to estimate that the sintered Cr-UO_2_ contains ~65% Cr^2+^ and ~35% Cr^3+^. The presence of Cr^3+^ in such a reducing environment can be explained by the existence of U^5+^ species in the calcined precursor material, but none in the corresponding sintered material (Fig. 3b, d). We hypothesise that Cr^3+^ originates from the reduction of U^5+^ to U^4+^ during sintering and the associated redox couple with Cr^2+^/Cr^3+^.

The aforementioned best fit model contained: 4.5 O atoms at a distance of 2.00 ± 0.01 Å; 1.5 O atoms at 2.44 ± 0.02 Å; 1 U atom at 3.34 ± 0.05 Å; 3 U atoms at 3.40 ± 0.07 Å; and 2 more U atoms at 3.91 ± 0.02 Å, indicating that a complex mixture of different Cr environments exists. Previous work has experienced some success in obtaining and analysing XAS data (both XANES and EXAFS^20,23^) on systems similar to this sintered sample. While informative, without underpinning understanding from simplified systems like the calcined powder investigated in the present study, and the lack of corroborating analysis from other techniques, resolution of the coordination environment of Cr from these previous XAS studies has proven challenging. As such, this proposed model, with supporting data from other techniques and fitting models grounded in DFT simulations, provides insight to a possible structure for sintered Cr-doped UO_2_. Given the complexity of the sample, including the likely multiple Cr oxidation states and coordination environments, further study of sintered materials developed under carefully controlled conditions should be performed, using this original analytical approach, to systematically evaluate the effect of sintering on the structure of Cr-UO_2_ materials.

### Mechanism of Cr incorporation in UO_2_

Considering the data presented, the following mechanism of Cr incorporation in sintered UO_2_ is proposed (Fig. 1c): Cr^2+^, already dissolved in the UO_2_ lattice (substituted onto U^4+^ sites and associated with O_v_) in the calcined starting material, is retained within the matrix upon sintering. For any given material where the proposed solubility limit of Cr is exceeded (> 700–1200 ppm Cr in UO_2_^3,6,9,11,15^), Cr is likely present in interstitial sites^8^ and is, thus, unbound and easily mobilised during sintering. This portion of Cr diffuses towards the grain boundaries allowing precipitation or volatilisation to occur (Fig. 1a). While it is not possible to confirm the oxidation state of this mobile fraction of Cr, it is likely that this reflects a combination of Cr^2+^, which was determined to exist in more than one environment, and some Cr^3+^, which was necessary to model the EXAFS data. The presence of Cr^3+^ is in agreement with µ-XANES analysis of an industrially-synthesised Cr-doped UO_2_ pellet^19^, which confirmed that precipitates of Cr comprised of Cr^3+^.

This work provides direct experimental evidence that, prior to high temperature heat treatment, the fundamental lattice structure of Cr-doped UO_2_ incorporates Cr^2+^ substituted on a U^4+^ site, charge balanced by the formation of both U^5+^ and O_v_ defects. The latter defects appear to remain upon sintering in a reducing atmosphere, while U^5+^ is reduced, promoting the oxidation of Cr^2+^ to Cr^3+^, although the main Cr oxidation state in these materials remains Cr^2+^. Were the materials to be sintered under an atmosphere with a higher partial pressure of oxygen, it is possible that Cr could be fully oxidised to Cr^3+^. Realisation of this mechanism not only provides detailed understanding of the defect structures in doped UO_2_ materials but can also be utilised to predict their behaviour in reactor, long-term storage and disposal conditions. The difficulty in realising the full local structure of aliovalent doped-UO_2_ materials, particular after the application of high temperature heat treatment, is highlighted. Such complexity can be, at least in part, resolved using high resolution spectroscopy techniques, which can be used to scientifically underpin the development of advanced doped uranium nuclear fuels. This work has shown that underpinning the dopant incorporation mechanism in calcined material is a fundamental step required to determining the evolution of crystal chemistry in Cr-doped UO_2_ fuels.

## Methods

### Sample preparation

A suite of Cr-doped UO_2_ samples were prepared *via* a wet synthesis method using uranium (VI) nitrate hexahydrate in solution (0.3 mol L^−1^) and chromium (III) nitrate nonahydrate in solution (1.6 mol L^−1^) (Cr(NO_3_)_3_∙9H_2_O 99.99 %, Sigma Aldrich). Concentrated ammonium hydroxide solution (5 mol L^−1^) was added at room temperature while stirring until a pH of 8–10 was reached, and a yellow precipitate was observed. Successful co-precipitation of U and Cr was confirmed by analysis of the supernatants by ICP-OES (ThermoFisher iCAP Duo6300), where 99.9 % precipitation for both U and Cr was achieved. Following vacuum filtration, precipitates were washed in deionised water and dried overnight at 90 °C to eliminate any remaining hydroxide. Thermal treatment at 750 °C for 4 h under a reducing (95% N_2_ / 5% H_2_) atmosphere allowed conversion of the precursor to oxide, confirmed by power x-ray diffraction analysis (employing LaB_6_ standard), which showed the fluorite crystal structure in all of the pure and Cr-doped UO_2_ samples (Supplementary Fig. 5). Homogeneity and powder reactivity were increased by milling at 35 Hz for 15 min. A stainless steel die was then used to uniaxially press 6 mm green pellets, at 500 MPa, before sintering in a reducing atmosphere (95% N_2_/5% H_2_) at 1700 °C for 8 h to obtain the final sintered pellets.

The concentration of Cr in the doped UO_2_ was verified by complete digest in concentrated nitric acid. Approximately 20 mg of calcined material was dissolved in 2 M HNO_3_ (ultrapure) and each vessel was magnetically stirred at 90 °C to accelerate the dissolution. An aliquot was analysed for the Cr concentration using inductively coupled plasma mass spectroscopy (ICP-MS, ThermoFisher iCAP RQ). Triplicate samples were measured to obtain an average Cr concentration for each sample. This method was repeated for sintered pellet samples, which were crushed to a powder using a pestle and mortar before being dissolved. The average Cr concentration is reported as Total Cr concentration in Fig. 1a where the difference between Calcined powder and Sintered pellet concentration is considered loss of Cr due to volatilisation during heat treatment.

### Electron probe microanalysis

Sintered pellets were ground using SiC paper, polished to 1 μm using diamond suspension and measured at the EPMA facility, School of Geosciences, University of Edinburgh, UK. A Cameca SX100 micro analyser with a 15 keV accelerating voltage, 160 nA beam current and a beam diameter of 1 μm was used with standards of FeCr_2_O_4_ and UO_2_. Elemental composition was analysed by wavelength dispersive X-ray analysis using crystals of PET and LPET to measure U Mα and Cr Kα signals. Measurements were taken at 15 points across the pellet surface and an average wt. % Cr within the UO_2_ matrix calculated as well as maps of 100 × 100 μm. The average wt. % Cr is reported as the Matrix Cr content in Fig. 1a and is considered the concentration of Cr in solid solution. The limit of detection was 25 ppm Cr hence the first two samples in the series could not be measured and are displayed as 0 ppm Matrix Cr content in Fig. 1a. The difference between Total Cr in Sintered pellets (as measured by ICP-MS and described above) and the Matrix Cr is considered the proportion of Cr present in precipitates (Fig. 1a and Supplementary Fig. 4).

MATLAB software was used to process images and adjust the threshold, allowing any areas of high Cr concentration to be observed. Back scattered electron images were overlaid with EPMA Cr elemental maps to highlight location of Cr.

### Raman spectroscopy

The crystal structure of calcined and sintered Cr-doped UO_2_ was analysed by Raman spectroscopy using a Renishaw inVia Reflex confocal spectrometer equipped with a Leica DM2500 microscope. Pressed green pellets of calcined powders and sintered samples polished to 1 μm using diamond suspension were measured using a 514.5 nm green argon laser with a holographic grating of 1800 lines mm^−1^ to acquire a spectral acquisition between 200 and 800 cm^−1^. The acquisition time was 30 s per spectrum and the laser power was set to 5 mW to limit sample oxidation. These configurations allowed a 2–3 cm^−1^ spectral resolution. An average of 10 point measurements across the surface of each sample, avoiding grain boundaries, was taken to confirm their reproducibility and homogeneity of the composition. Information from the Raman spectra was extracted using Lorentz function fitting where the intensity ratios of the triplet defect peaks (U1, U2 & U3) relative to the main UO_2_ T_2g_ band (445 cm^−1^) was carried out. These defect peaks correspond to O_v_ (U1) (~527 cm^−1^), LO mode (U2) (~574 cm^−1^) and O_i_ (U3) (~634 cm^−1^)^31^.

### X-ray absorption spectroscopy

XAS measurements were performed, at room temperature, on both calcined powder and sintered Cr-doped UO_2_ at several beamline facilities. All samples diluted with poly-ethylene glycol (PEG) to 1 absorption length for the desired element and edge (Cr K- or U M_4_-edges). Fluorescence mode was employed at beamline I20-scanning at DLS to measure the Cr K-edge (5989 keV) using a 64-element Ge detector with Xspress4 signal processing. The beam size was 400 × 300 µm (FWHM). The wiggler-sourced I20 beamline utilises a Si (111) four-bounce crystal monochromator, which results in high flux and excellent energy resolution. As such, I20-scanning is uniquely suited to determine the local structure of Cr-doped UO_2_, especially where Cr was in low concentration in the heavily absorbing UO_2_ matrix. Multiple scans were taken to improve data quality with energy steps of 5 eV (5889–5985 eV), 0.2 eV (5985–6010 eV) with a time step of 1 s step^−1^ and 0.04 Å^−1^ (6010-6700 eV) with a time step of 1–6 s step^−1^ in this regions. High-energy resolution fluorescence detection X-ray absorption near edge spectroscopy (HERFD XANES), at the U M_4_-edge (3728 eV), was carried out at the BM20 beamline at the European Synchrotron (ESRF)^32^, France, with a help of X-ray emission spectrometer^33^. Surfaces of as-sintered pellets were measured in fluorescence mode, while calcined powders were mixed with poly-ethylene glycol (PEG) and pressed into 6 mm pellets for analysis, also in fluorescence mode. Standards of known U and/or Cr valence state and coordination, including UO_2_, CrUO_4_, Cr_2_O_3_ and Cr Foil were also measured, in transmission and fluorescence mode to account for self-absorption effects.

A standardised element foil was measured simultaneously with Cr-doped UO_2_ samples and used as a reference to which the *E*_0_ positions of all raw data was aligned. A cubic spline background subtraction and normalisation procedure^34^ was carried out on all aligned data before multiple scans were merged using Athena^35^. The EXAFS region, measured in eV, was then converted to wavenumber, k, with units of Å^−1^. The Fourier Transform of this data was then fit using models generated from applying possible scattering paths of the central absorber (Cr) to surrounding atoms (O and U). Scattering paths were generated from appropriate structural CIF files, using the FEFF 6 algorithm^36^ in Artemis. During the process of trialling a range of possible EXAFS models, a number of parameters; range of k (Å^−1^) and radial distance r (Å), and the amplitude reduction factor (S_0_^2^) were optimised for the data and the following parameters allowed to refine; degeneracy (N), fitted bond length (R)(Å), shift from Cr K-edge position (5989 keV) (∆*E*_0_) and the Debye–Waller factor (σ^2^). Ultimately, the final fits fixed the degeneracy (N) to the values from the relevant CIF files, or a manual refinement was performed. The F-test was applied to each fitted path, the result (α) indicated the confidence of adding the path to improve the fit (>67% gives a confidence of 1σ, >95 % gives a confidence of 2σ)^37^. The bond valence sum (BVS) returns a summation of bond distances in a coordination shell, which is equal to the formal oxidation state of the cation absorber^38^. The bond valence of Cr-O coordination was calculated for both calcined and sintered material and used to determine the proportion of Cr^2+^/Cr^3+^ in the sintered material.

Despite efforts during collection of Cr K-edge data, reduced resolution of the XANES region at the lowest Cr concentrations resulted in inconsistency in the background removal and normalisation, most prominently in the 300 ppm sample for both oxide powder and sintered samples (Fig. 2c, e and Supplementary Data Fig. 6). Nevertheless, the same features were observed for all materials and, as such, only the samples with the highest dopant concentrations were utilised for Cr K-edge EXAFS analysis.

Principal component analysis was carried out on U M_4_-edge data (raw data was pre-processed in Athena as described above) as part of the Iterative Target Transformation Factor Analysis^39^ to calculate the relative concentration of each component of the absorption spectra; UO_2_ and CrUO_4_ standards were used.

### X-ray diffraction

Powder XRD (p-XRD) characterisation was performed using a PANalytical Xpert3 diffractometer in reflection mode with a 45 keV/40 mA generator. To avoid oxidation, the calcined oxide samples were measured immediately post-heat treatment (i.e. within 10 min of removal from the furnace). Similarly, sintered samples were crushed using a pestle and mortar upon removal from the furnace, within a controlled inert atmosphere (N_2(g)_), and immediately measured. Data were collected between 5° and 100° 2θ with a step size of 0.013° and a step time of 40 s, a fixed slit size of 0.5 was used. An internal standard of LaB_6_ (20–30 wt. %) was used for data alignment, and p-XRD patterns corrected in the WinXPow software. Le Bail refinements carried out in the Topas software, allowing accurate determination of the lattice parameter as a function of Cr content in UO_2_.

## Supplementary information

Corkhill_PR File

Supplementary Information

## Data Availability

The data that support the findings of this study are available from the corresponding author upon request.
