# Supplementary material for: Cr2+ solid solution in UO2 evidenced by advanced spectroscopy
Source: Commun Chem. 2022 Dec 1;5:163. doi: 10.1038/s42004-022-00784-3 (PMC9814952; doi:10.1038/s42004-022-00784-3)
Supplement: Supplementary file 2 — Supplementary Information [file 42004_2022_784_MOESM2_ESM.pdf]

Cr^{2+} solid solution in UO_2 evidenced by advanced spectroscopy

Hannah Smith[1], Luke T. Townsend[1], Ritesh Mohun[1], Théo Cordara[1], Martin C. Stennett[1], J. Frederick W. Mosselmans[2], Kristina Kvashnina[3,4], Claire L. Corkhill[1*]

[1] NucleUS Immobilisation Science Laboratory, Department of Materials Science and Engineering, The University of Sheffield, Sheffield, UK.
[2] Diamond Light Source, Harwell Science and Innovation Campus, Didcot, UK.
[3] Helmholtz-Zentrum Dresden-Rossendorf (HZDR), Institute of Resource Ecology, PO Box 510119, 01314, Dresden, Germany.
[4] The Rossendorf Beamline at ESRF – The European Synchrotron, Grenoble, France.

*Corresponding Author: c.corkhill@sheffield.ac.uk

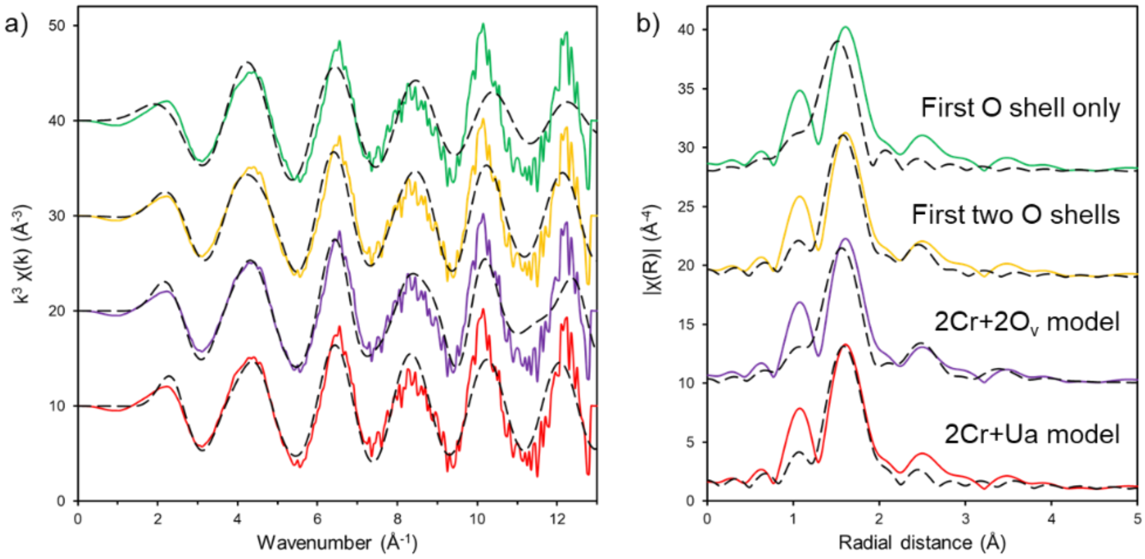

Supplementary Figure 1. Comparison of a selection of different fitting models for the Cr-doped UO_2 calcined powder (2400 ppm) trialled during EXAFS modelling. a k^3-weighted Cr K-edge spectra fit between 3-11.5 and; **b** the associated Fourier transform fit between 1.35 - 3 for the 'First O shell only' and 'First two O shells', and '2Cr+2O$_v$ model' and '2Cr+Ua model' were fit between 1.35 – 3.9.

Supplementary Table 1. EXAFS fitting parameters for the different fitting models shown in Supplementary Figure 5 for Cr-doped UO_2 calcined powder (2400 ppm). 'First O shell only' and 'First two O shells' utilised the 1Cr + $1O_V$ CIF file used in the main manuscript. 'First O shell only' fit acts as a direct comparison with the EXAFS fitting model employed in Mieszczynski *et al.* (2014). '2Cr+$2O_V$' and '2Cr+Ua' models are derived from other CIF files from the work by Sun *et al.* (2020). (S_0^2) is the amplitude reduction factor, (ΔE_0) the shift from Cr K-edge position (5.989 keV), (N) the degeneracy, (R) (Å) the fitted bond length, and (σ^2) the Debye-Waller factor.

	First O shell only	First two O shells	2Cr+$2O_V$ model	2Cr+Ua model
S_0^2	0.90	0.90	0.90	0.90
ΔE_o	-7.7(43)	-1.8(22)	-3.1(24)	-0.1(35)
N (Cr-O1)	6	4	5	4
R(Cr-O1)	1.97(2)	1.99(1)	1.99(1)	2.00(2)
σ^2 (O1)	0.005(2)	0.001(1)	0.003(1)	0.001(1)
N (Cr-O2)	-	3	2	8
R(Cr-O2)	-	2.96(4)	-0.001(3)	0.027(25)
σ^2 (O2)	-	0.005(6)	2.93(3)	3.92(17)
N (Cr-U1)	-	-	4	4
R(Cr-U1)	-	-	3.54(6)	2.74(18)
σ^2 (U1)	-	-	0.017(9)	0.056(50)
N (Cr-U2)	-	-	2	-
R(Cr-U2)	-	-	3.83(11)	-
σ^2 (U2)	-	-	0.017(15)	-
N (Cr-Cr1)	-	-	-	1
R(Cr-Cr1)	-	-	-	2.51(7)
σ^2 (Cr1)	-	-	-	0.016(12)
N (O1-U1 MS)	-	-	-	16
R (O1-U1 MS)	-	-	-	3.34
σ^2 (O1-U1 MS))	-	-	-	0.057
R-factor	0.145	0.034	0.041	0.039
Bond valence sum (total)	3.172	2.070	2.574	1.925

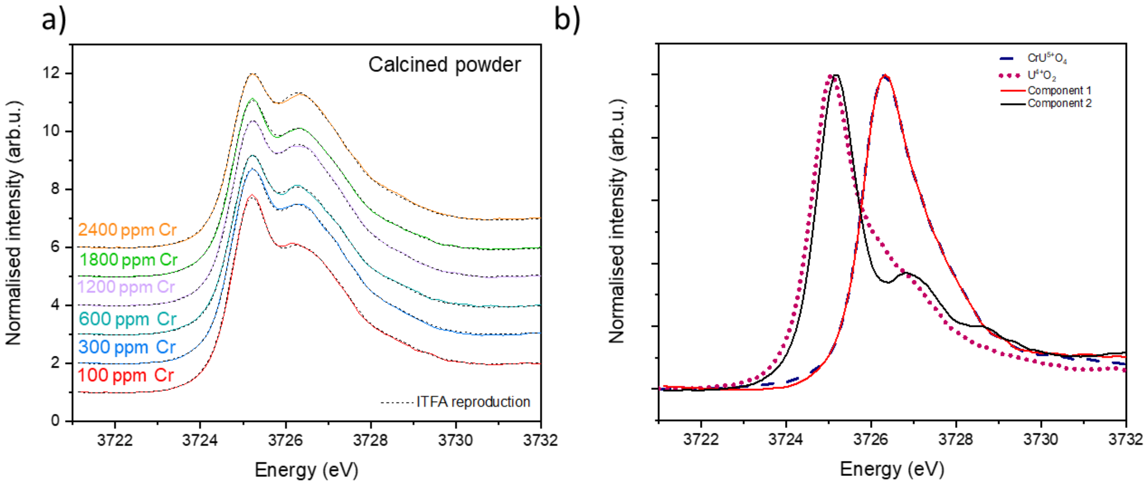

Supplementary Figure 2. HERFD XANES U M_4 edge spectroscopy analysis of calcined material. **a** ITFA reproductions of Cr-doped UO_2 calcined powder using only two components. Coloured lines are the original data and black dotted lines are the reproduced spectra by ITFA and; **b** Comparison between the components produced during the PCA analysis using ($CrU^{5+}O_4$) and $U^{4+}O_2$ standards.

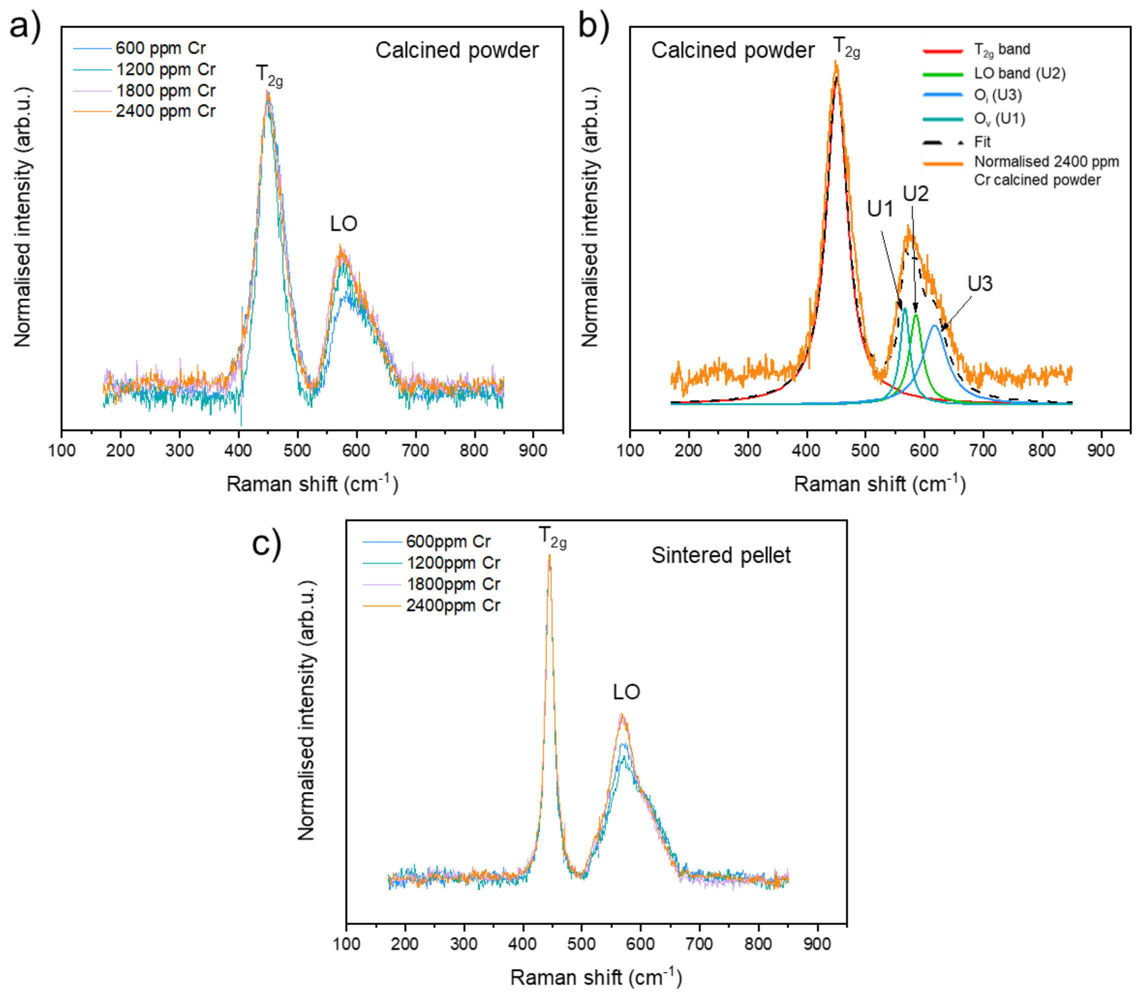

Supplementary Figure 3. Raman spectra for Cr-doped UO_2 calcined and sintered material. **a** Calcined powder Raman spectrum as a function of Cr content; **b** deconvolution of 2400 ppm Cr-doped calcined powder; **c** sintered pellet Raman spectra.

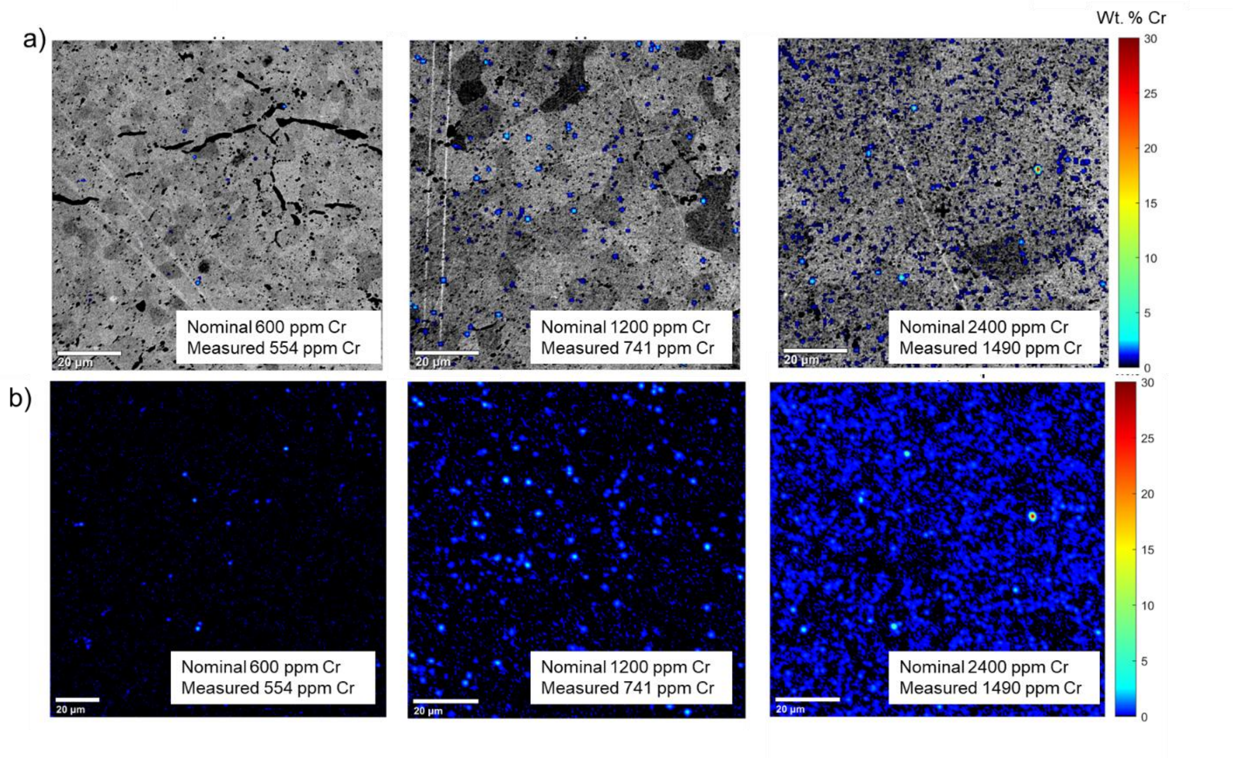

Supplementary Figure 4. Distribution of Cr in Cr-doped UO_2 sintered pellets measured by EPMA. a SEM micrographs overlaid with Cr elemental distribution maps showing distribution of Cr as precipitates at grain boundaries and within the UO_2 matrix; **b** Cr elemental distribution maps.

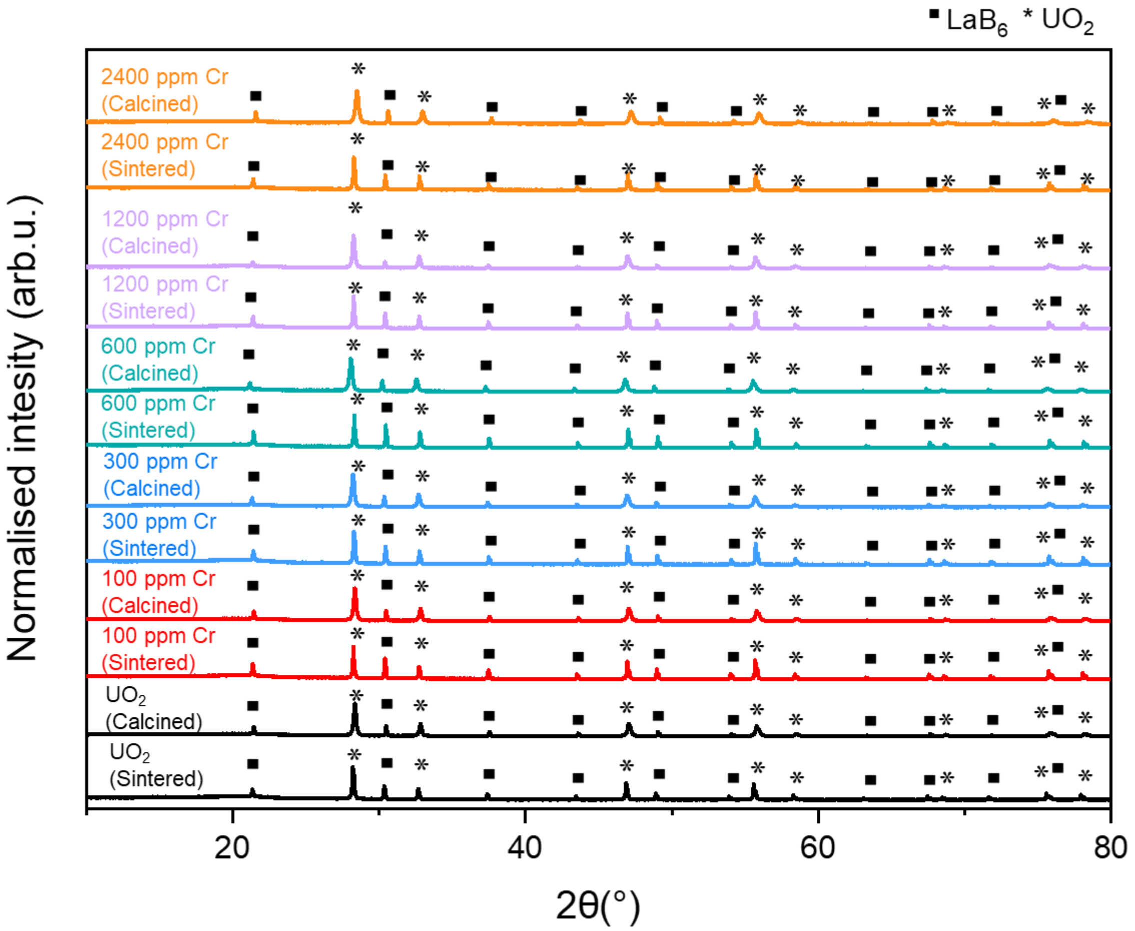

Supplementary Figure 5. P-XRD of post heat treatment, calcined and sintered material normalised using LaB_6 internal standard.

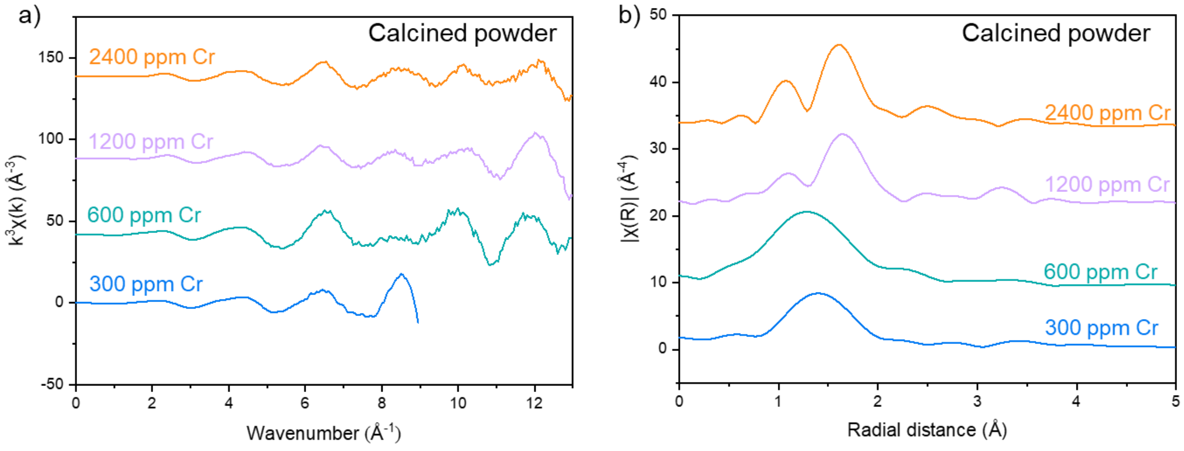

Supplementary Figure 6. Comparison of Cr K-edge data of Cr-doped UO_2 calcined powder (300 – 2400 ppm Cr). a k^3-weighted Cr K-edge spectra (K-space) and; **b** the Fourier transform (R-space).

Supplementary Table 2. Cr K-edge XANES positions derived from the peak of the first derivative and 0.5 a.u. of the normalised edge step.

Category	Sample	Peak of the first derivative (eV; ± 0.1)	0.5 a.u. of the normalised edge step (eV; ± 0.1)
Standard	$Cr(III)_2O_3$	6006.0	6000.3
	$Cr(II)UO_4$	6003.2	6001.5
	Cr(0) Foil	5989.0	5994.0 – 5995.7
Calcined	300 ppm Cr	6005.5	6002.9
	600 ppm Cr	6003.2	6000.5
	1200 ppm Cr	6003.2	6000.8
	2400 ppm Cr	6003.7	6000.6
Sintered	300 ppm Cr	6000.4	5995.0
	600 ppm Cr	6000.2	5995.1
	1200 ppm Cr	6000.0	5995.4
	2400 ppm Cr	6000.1	5995.7
