# Supplementary material for: Cr2+ solid solution in UO2 evidenced by advanced spectroscopy
Source: Commun Chem. 2022 Dec 1;5:163. doi: 10.1038/s42004-022-00784-3 (PMC9814952; doi:10.1038/s42004-022-00784-3)
Supplement: Supplementary file 1 — Corkhill_PR File [file 42004_2022_784_MOESM1_ESM.pdf]

nature portfolio

Peer Review File

Cr2+ solid solution in UO2 evidenced by advanced spectroscopy

Open Access This file is licensed under a Creative Commons Attribution 4.0 International License, which permits use, sharing, adaptation, distribution and reproduction in any medium or format, as long as you give appropriate credit to the original author(s) and the source, provide a link to the Creative Commons license, and indicate if changes were made. In the cases where the authors are anonymous, such as is the case for the reports of anonymous peer reviewers, author attribution should be to 'Anonymous Referee' followed by a clear attribution to the source work. The images or other third party material in this file are included in the article's Creative Commons license, unless indicated otherwise in a credit line to the material. If material is not included in the article's Creative Commons license and your intended use is not permitted by statutory regulation or exceeds the permitted use, you will need to obtain permission directly from the copyright holder. To view a copy of this license, visit http://creativecommons.org/licenses/by/4.0/.

Reviewers' comments:

Reviewer #1 (Remarks to the Author):

The study on the Cr incorporation in UO2 using spectroscopic studies carried out by Smith discusses one of the important scientific subject areas that is related with nuclear fuels. Dopants such as Cr can produce enlarged UO2 grains that help reduce fission gas release during reactor operation and safeguard the nuclear fuel core. Since there are some gaps and discrepancies in the reported literature on the mechanism and chemistry of the Cr-doped UO2 system due to its complexity, such systems need to be studied using advanced techniques like what is presented here. Therefore, I suggest publishing this manuscript in Communications Chemistry journal after the authors consider the following comment.

The conclusions presented in this manuscript are supported by the experimental advanced spectroscopic techniques, which I believe a good addition to the literature for this system. The only comment I have for the authors to consider is the use of N2-based cover gas for the sintering at high temperature 1700°C. It is shown that some oxides (e.g., ZrO2) react with N2 cover gas making oxynitrides at such high temperatures. If 'N' is added to the Cr-doped UO2, the data presented here for the sintered samples may have affected by it. Therefore, I would suggest the authors to review their data (especially, XRD and SEM/EDS) to verify that cover gas was not introduced into the oxide lattice.

Line 197 and 206 – 218: What was the reason for the use of N2-based cover gas as the reducing environment? I am also concerned that some of oxygen or oxygen vacancy sites might have replaced by nitrogen at the sintering temperature. Did you compare the XRD patterns of calcined and sintered samples for the presence of minor secondary phases in the sintered samples, peak doublets, peak broadening or any other defects such as amorphous characteristics? Could the greater LP of the sintered samples be a result of nitrogen incorporation into the oxide lattice? Presenting profile fits of the XRD patterns of sintered samples in the supplemental data can be helpful in this verification.

Reviewer #2 (Remarks to the Author):

[Editorial Note: Please see attachment.]

Reviewer #3 (Remarks to the Author):

The paper submitted by Smith et al. provides a very interesting study addressing the redox speciation of chromium and uranium in Cr-doped UO2 nuclear fuels. Although studied for long to enhance grain growth during sintering, the exact mechanism of chromium incorporation remains widely unknown, even if it is of primary importance to forecast the long-term behaviour of such materials after their use in-pile.
From a general point of view, the manuscript is written in a clear and concise style. Also, the conclusions are supported by a large number of spectroscopy experiments, including XAS measurements. It is important to recall that such data are scarce on radioactive materials and almost deserve publication by themselves !
To be short, my feeling is that this manuscript should be published provided some minor modifications, as suggested below :

- axis captions on the figures are very small and might he bardly readable once inserted in the final version.

- I'm a little bit confused with equation 1 (line 168) : here what is oxidizing uranium ? More generally,

do the authors have any idea about the Uranium redow speciation in the starting powder ? Indeed, if uranium only exists as U(IV) and chromium as $Cr2+$ before sintering, I do not clearly understand how can U(V) can form. Also, it is not clear to me what is driving the final amount of U(V) in the samples, which seems to be undependant on the Cr doping rate. Does that means that the amount of chromium really incorporated in the $UO2$ structure is always the same, and that excess of Cr further exists as metallic precipitate ? Maybe these points should be addressed in more details to be more understandable.

- could you be more specific about the Raman aquisition conditions on lines 363-374 ? Notably, please add laser power and aquisition time considered

- line 408 : there is a typo in 'parameter'

This study aims to investigate the chemical state and speciation of solute Cr in Cr-doped UO2 fuels (DF). Combining EXAFS and FEFF modeling results, authors have claimed a chromium(2+) uranium substitutional model is the main solution mechanism. Although the subject is worthy of investigation, primarily because substantial discrepancies exist in the literature [as also stated in the manuscript, line 29] regarding the chromium local environment (oxidation state and chemical distribution when under soluble form) in DF, this study fails to bring new experimental and EXAFS-modelling insights:

Page 3 lines 88: When discussing the results reported in Ref. [23], the authors have mentioned "…….the data quality reported in Ref. 23 was not sufficient to obtain reliable evidence for the Cr speciation and local environment". The reviewer is not convinced, if this was the case, when the EXAFS oscillation spectrum and the corresponding FT-EXAFS spectrum in Fig. 2 (k-3 weighted data, up to k-range 13) in the manuscript are compared with those results reported in Ref. 23 (Fig. 3., k-2 weighted data, k-range up to 12).

Page 8, line 252: When discussing the FT-EXAFS data and modeling using FEFF paths, the authors have stated "Previous work has experienced limited success in obtaining and analyzing XAS data (both XANES and EXAFS) on systems similar to this sintered sample. As such, this proposed model, generated using high flux, low-noise scanning EXAFS, provides insight to a possible structure for sintered Cr-doped UO2." Here, authors have cited three references: [Ref. 20, XANES data measured at ESRF using 300 micron beam spot, DF contains Cr2O3 precipitates, area probed 600 micron diameter disc], [Ref. 21, new experimental data is not reported by the authors, used the data published in Refs. 20 and 23, re-evaluated the XANES data, and unexpectedly found Cr(2+) species…] and [Ref. 23, data measured at SLS using 2 micron beam spot, DF contains Cr2O3 precipitates, …..]. It is found that in Ref. [23] the curve fitting result of multiple shell analysis is not reported and those authors have discussed what they believe. Therefore, the reviewer does not acknowledge the aforementioned statements. The reviewer is also aware that high flux, low-noise scanning EXAFS experiments are routinely performed at the ESRF and SLS.

RESULTS OF THIS MANUSCRIPT: EPMA results [supplementary Fig. 3 (sintered pellets)] show that the prepared UO2 materials contain solute Cr and insoluble Cr in the form of submicron-sized second phase precipitate (SPP, Cr2O3 phase used in FEFF calculations). Regarding the calcined powder samples the authors have claimed on page 3 line 111, "All oxide powders were shown to contain the intended concentration of Cr when fully digested (in the range 300 to 2400 ppm)". It has been also stated in the manuscript (on page 6 line 173) "Given that the concentrations of Cr in the calcined material exceed the generally agreed solubility limit of Cr in UO2 reported to be in the range of 700 – 1200 ppm, it can be assumed that any excess Cr is present as positive interstitial defects". The statements made are contradictory. However, the authors do not consider other possibilities, such as, local clustering of excess Cr (although unlikely, as mentioned on page 8 line 236 in the manuscript) and/or the presence of ultrafine chromium oxide particles in the calcined samples.

Cr K-edge XAS measurements are performed at the DLS using X-ray beam size of 400 micron. This means, apart from solute Cr in the analyzed materials, a large number of SPP located within the probed volume were also illuminated by the incident light and contributed to the measured Cr K-edge XAS spectra. This raises question on the reported FEFF modelling of measured EXAFS data for the calcined oxide powder, where any Cr contribution of precipitated Cr (Cr2O3 or any other possible form) has been neglected in the data analysis. How the authors know that all Cr are only in solid solution when

the Cr-content have exceeded the solubility limit of Cr? Multiple ultrafine Cr-containing particles (in oxide phase or other form) could (and are likely to) be present in the materials. These points are not addressed in the paper.

XANES data: In this study, conclusions are ascribed to the effect of Cr oxidation state(s) to describe the chromium incorporation mechanism. However, no results of the detailed XANES analyses (first or second inflection point, first or second-derivative procedure, etc.) are provided in the manuscript. The assignment of oxidation number (+2) of all solute Cr is based only on the observed pre-edge feature in the XANES data. It is not a new result, previously reported in the literature. The Cr(2+) assignment, from the reassessment of the published XANES data reported in Ref. [21], is not fairly convincing [the spectral broadening due to the core-hole lifetime at the Cr K edge > 1 eV].

EXAFS data: The best fit of the FT-EXAFS data, measured from 2400 ppm Cr-containing calcined powder sample, was achieved by utilizing a 1Cr(2+)+1Ov model structure. This model is based on the assumption that most of the Cr atoms (say between 700 and 1200 ppm; as mentioned on page 6 line 175 in the manuscript) are dissolved in UO2, the material is precipitate free, and the authors think (or assume) that excess Cr is present as positive interstitial defects (line 175 in the manuscript) as predicted using DFT-based calculations [(Ref. 8 in the manuscript); to the best of this reviewer's recollection, there is no experimental result, reporting interstitial Cr content in DF, available in the open access literature]. However, the authors do not consider the contribution of those interstitial Cr atoms in their FT-EXAFS data analysis. Secondly, U(5+) has been identified in this sample [HERFD-XANES results of as-produced calcined material (Fig. 3b)], and the authors did not check a lattice model in its simplest form of 1Cr(3+)-U(4+) structure that can be directly related to compensate the charge imbalance when Cr(3+) is substituted for U(4+).

For the EXAFS data of sintered pellet (2400 ppm Cr containing material), the best fit was achieved by utilizing the 1Cr(2+)+1Ov structure plus a 2Cr(2+)+2Ov structure for solute Cr, and a Cr3+ environment of Cr(3+)2O3 phase. In the manuscript (line 235) the authors have stated that EXAFS data analysis for this sample was challenging, determined the local coordination environment of chromium ions in the sample, and presented a complex structural model for solute Cr(2+) (65%) [35% is Cr(3+) of Cr2O3 phase] present in the sintered DF pellet in its final form. However, the authors do not discuss or report any structural analyses of other possible structure candidates [such as, 2Cr(3+)+1Ov for solute Cr, a mixed 2Cr(3+)+1Ov and Cr(3+)2O3 model, ..] that have been also suggested in the literature.

EXAFS results and structural parameters reported in Table 1: The local atomic environment around Cr atoms, in calcined DF powder and sintered DF pellet, is examined by analyzing the EXAFS part of the XAS data. A multi-shell fitting approach has been employed for data fitting: four coordination shells (Cr-O1, Cr-O2, Cr-U1 and Cr-U2) for the calcined powder sample (containing 2400 ppm Cr); and five coordination shells (Cr-O1, Cr-O2, Cr-U1, Cr-U2 and Cr-U3) for the sintered sample (containing 2400 ppm Cr). All fits used the site-selective three (?) free parameters (N, R, and sigma) contained in each single-scattering FEFF path included in the data analysis. Knowing the fact that the said parameters are strongly correlated in the EXAFS equation: Then, (a) the EXAFS-derived numerical values of structural parameters reported in Table 1 are questionable since the total number of free parameters are 12 and 15 (+ additional FEFF paths taken for the undissolved Cr2O3 phase), respectively, for the two experimental data-set analyzed; (b) reason for the assumption of an overall delta-E0 (-0.80 eV for the calcined powder and -6.0 eV for the sintered sample) to be the same for both Cr-O1(or O2) and

Cr-U1(or U2 and U3) scatterings paths is not clear, and why this parameter was constrained during the final refinements; (c) most importantly, how the authors know that multiple-scattering (MS) effects should be excluded [(MS) FEFF paths] in their data analysis. The authors have neglected the MS contribution in their data analysis, and do not provide any justification for the same in their manuscript; (d) although the complex structure of the FT-EXAFS spectra (Fig. 2b and Fig. 2e) indicates a structurally complicated Cr local environment in the samples, a shell-by-shell fit of Fourier-filtered data was not attempted by the authors; (e) assuming that the strong peak near 1.5 A in Fig. 2b (calcined sample) and 2e (sintered sample) is due to the first coordination Cr-O shell, the authors did not assign the visible FT peak near 1 A in the data (Fig. 2b) that is, however, absent in Fig. 2e; (f) in crystalline UO2, the coordination number for the first U-O shell is 8. According to the fit results of both samples in Table 1, the coordination number of 7 has been determined and the Cr-O bond length distribution is bimodal in the immediate neighborhood of the dopant ions. However, for the second coordination Cr-U shell [equivalent U-U, N=12, crystalline UO2], the fit results of DF materials show highly undercoordinated chromium sites with only 6 U atoms [N=6 [(Cr-U1)+(Cr-U2)+(Cr-U3)]] instead of 12 U atoms, and the situation corresponds to a trimodal distribution of bond lengths. The origin of these anomalies of the atomic coordination parameters are not discussed in the paper.

The authors did not clarify the aforementioned points in their manuscript. A second and closely related problem is that the presentation is incomplete and it is difficult, sometimes impossible, to understand what has been done. It is not clear from the paper, exactly what the authors have learned and what their objective is for the presentation. Consequently, the reviewer advises to reject the paper.

Reviewer Comments Rebuttal

In this document, we have listed the Reviewer comments with our corresponding responses directly below, written in blue. Any changes that have been made to the manuscript are made in the response document in red, and highlighted in the revised manuscript in blue text.

Reviewers 1 and 3 suggest publication after minor revisions, which we have made. Reviewer 2 suggests reject, on the basis of the fact that our EXAFS data do not, in their opinion, bring any new insight to the crystal chemistry of Cr-doped UO_2. We assert that they have come to this conclusion because, in our attempt to write a brief communication-style paper, we omitted details of our attempts to fit our EXAFS data with models that were previously proposed in the literature. In our revised manuscript we have now added this information, which shows that the previous models do not resolve the structure as well as the one that we propose. Undoubtedly their comments have allowed us to make additions to the text and supplementary information that strengthen our reasoning.

We hope that these additions are sufficient to allow the editor to make a decision regarding our manuscript without further review; however, if Reviewer 2 does re-review our paper, we hope that they are willing to accept our model presents a statistically relevant alternative view point on the crystal chemistry of Cr-doped UO_2, underpinned by data from multiple techniques and using a simplified system to avoid the challenges of interpreting complex real nuclear fuels.

Reviewer 1

General Comments: The study on the Cr incorporation in UO_2 using spectroscopic studies carried out by Smith discusses one of the important scientific subject areas that is related with nuclear fuels. Dopants such as Cr can produce enlarged UO_2 grains that help reduce fission gas release during reactor operation and safeguard the nuclear fuel core. Since there are some gaps and discrepancies in the reported literature on the mechanism and chemistry of the Cr-doped UO2 system due to its complexity, such systems need to be studied using advanced techniques like what is presented here. Therefore, I suggest publishing this manuscript in Communications Chemistry journal after the authors consider the following comment. The conclusions presented in this manuscript are supported by the experimental advanced spectroscopic techniques, which I believe a good addition to the literature for this system.

Author Comment: We thank the Reviewer for their positive assessment of our study, the identification that the work is a good addition to the current literature, and for their recommendation that the article should be published following the address of their single comment.

Comment 1: The only comment I have for the authors to consider is the use of N_2-based cover gas for the sintering at high temperature 1700°C. It is shown that some oxides (e.g., ZrO_2) react with N_2 cover gas making oxynitrides at such high temperatures. If 'N' is added to the Cr-doped UO_2, the data presented here for the sintered samples may have affected by it. Therefore, I would suggest the authors to review their data (especially, XRD and SEM/EDS) to verify that cover gas was not introduced into the oxide lattice. What was the reason for the use of N_2-based cover gas as the reducing environment?

Response 1: We thank the author for raising this important consideration. During the EMPA analysis of our samples, we checked for any signal arising from N in our samples – there was none. Since this was the case, we did not measure element maps of N and cannot evidence our claim; however, there was no evidence for the presence of N in any of the other analytical techniques we used (e.g. absence of N-related signals in the Raman data).

Comment 2: I am also concerned that some of oxygen or oxygen vacancy sites might have replaced by nitrogen at the sintering temperature. Did you compare the XRD patterns of calcined and sintered samples for the presence of minor secondary phases in the sintered samples, peak doublets, peak broadening or any other defects such as amorphous characteristics? Could the greater LP of the sintered samples be a result of nitrogen incorporation into the oxide lattice? Presenting profile fits of the XRD patterns of sintered samples in the supplemental data can be helpful in this verification.

Response 2: As the Reviewer requested, we have added a figure to the supplementary information to show the XRD pattern fit (Supplementary Data Figure 4):

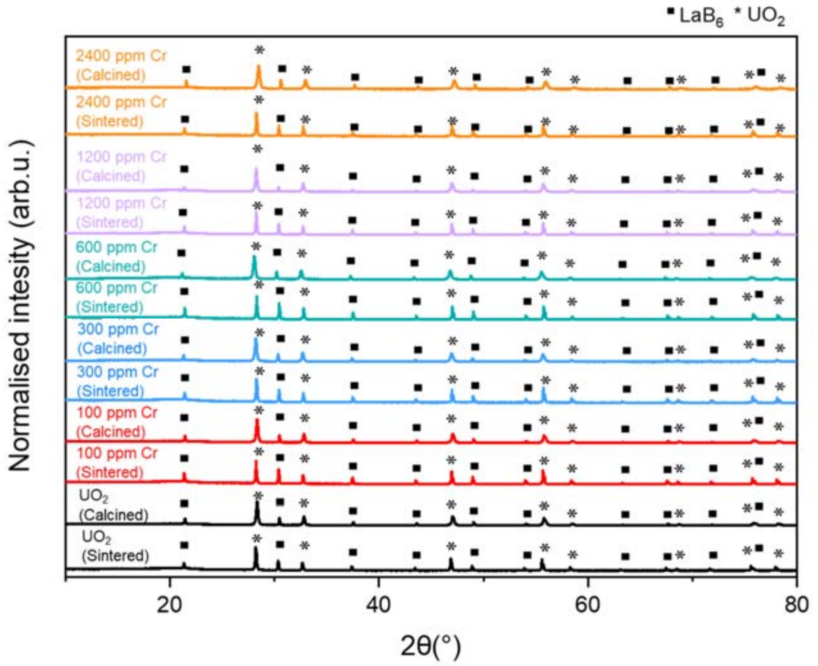

Supplementary Figure 4. P-XRD of post heat treatment, calcined and sintered material normalised using LaB_6 internal standard.

<u>**Reviewer 3**</u>

General Comments: The paper submitted by Smith et al. provides a very interesting study addressing the redox speciation of chromium and uranium in Cr-doped UO2 nuclear fuels. Although studied for long to enhance grain growth during sintering, the exact mechanism of chromium incorporation remains widely unknown, even if it is of primary importance to forecast the long-term behaviour of such materials after their use in-pile. From a general point of view, the manuscript is written in a clear and concise style. Also, the conclusions are supported by a large number of spectroscopy experiments, including XAS

measurements. It is important to recall that such data are scarce on radioactive materials and almost deserve publication by themselves!

Author Comment: We thank the Reviewer for their highly positive appraisal of our manuscript and note that they find it clear and concise. They specifically highlight the novelty of our XAS measurements and point out that the conclusions from analysis by other techniques are supported by our XAS interpretation.

Comment 1: Axis captions on the figures are very small and might he barely readable once inserted in the final version.

Response 1: We thank the Reviewer for raising this. We have revised the figures to make the text size more visible.

Comment 2:

- **a.** I'm a little bit confused with equation 1 (line 168): here what is oxidizing uranium?
- **b.** More generally, do the authors have any idea about the Uranium redox speciation in the starting powder? Indeed, if uranium only exists as U(IV) and chromium as Cr^{2+} before sintering, I do not clearly understand how can U(V) can form.
- **c.** Also, it is not clear to me what is driving the final amount of U(V) in the samples, which seems to be undependant on the Cr doping rate.
- **d.** Does that mean that the amount of chromium really incorporated in the UO2 structure is always the same, and that excess of Cr further exists as metallic precipitate?

Response 2: The reviewer refers to the following equation and we have broken their question into several parts to aid explanation.

$$yCr^{2+} + U^{4+}O_2 \rightarrow ((U^{4+}_{1-x}U^{5+}_{x})_{1-y}Cr^{2+}_{y})O_{2-\frac{y}{2}}$$

- **a.** It was our intention with this equation to define the stoichiometry of the Cr-doped UO_2 based on the knowledge acquired regarding the U^{5+} content (determined by U M_4-edge XANES) and presence of oxygen vacancies (determined by Raman spectroscopy). We understand the Reviewer's confusion: using a "reaction equation" may not be the best way to define the stoichiometry, since it implies the reaction of Cr^{2+} with UO_2 when, in fact, we hypothesise that it is the reaction of Cr^{3+} with UO_2 that forms Cr^{2+}-doped UO_2. This answers the first part of the Reviewer's question: the reduction of Cr^{3+} drives the oxidation of U^{4+} to U^{5+}. To avoid this confusion, we have removed the equation and amended the text as follows:

Lines 163 to 166: This corroborates the hypothesis that charge balance for Cr^{2+} within Cr-doped UO_2 is provided by both O_v and U^{5+} defect formation, with O_v potentially playing a more prominent role. The final stoichiometry is, therefore, $((U^{4+}_{1-x}U^{5+}_{x})_{1-y}Cr^{2+}_{y})O_{2-\frac{y}{2}}$.

- **b.** The starting calcined material, according to U M_4-edge XAS, was close to 100% U^{4+}. This is corroborated by the lattice parameter of the material (shown in Fig. 3f), which was determined to be 5.4655(1) Å. According to Elorrieta et al. (2016) (Phys. Chem. Chem. Phys. 18, 28209), this gives an oxygen stoichiometry very close to UO_2.
- **c.** Observation of Figure 3b shows that the U M_4-edge peak corresponding to U^{5+} increases somewhat with increased Cr content. We believe that U^{5+} is formed to charge balance Cr^{2+} (hence the small increase with increasing Cr content) but that this

is not the only mechanism. Indeed, on lines 163 to 165, we propose that both O_v and U^{5+} are likely defects arising from the requirement to charge balance Cr^{2+}. To make it clear that the increase in U^{5+} is small, and that perhaps O_v play a more dominant role in the charge balancing mechanism, we have made the following addition to the text:

Lines 163 to 165: This corroborates the hypothesis that charge balance for Cr^{2+} within Cr-doped UO_2 is provided by both O_v and U^{5+} defect formation, with O_v potentially playing a more prominent role.

d. Our hypothesis, explained on Lines 171 – 179 is that the solubility limit of Cr in UO_2, i.e., the ability of Cr^{2+} to sit on U^{4+} lattice positions, is much lower than reported in the literature. We say "Given that the concentrations of Cr in the calcined material exceed the generally agreed solubility limit of Cr in UO_2 (reported to be in the range of 700 – 1200 ppm[3, 6, 9, 11, 15]), it could be assumed that excess Cr may be present as positive interstitial defects.[8]" That is to say, once the U lattice positions have reached capacity w.r.t. atoms of Cr (charge balanced by the formation of U^{5+} and O_v, both of which increase in concentration with Cr content), excess Cr may sit within interstitial sites, charge balanced by O_i.

Comment 3: Could you be more specific about the Raman aquisition conditions on lines 363-374? Notably, please add laser power and aquisition time considered.

Response 3: The information has been added as requested (see Lines 363 to 368).

Comment 4: line 408: there is a typo in 'parameter'

Response 4: Amended.

<u>Reviewer 2</u>

General Comments: This study aims to investigate the chemical state and speciation of solute Cr in Cr-doped UO_2 fuels (DF). Combining EXAFS and FEFF modeling results, authors have claimed a chromium(2+) uranium substitutional model is the main solution mechanism. Although **<u>the subject is worthy of investigation</u>**, primarily because substantial discrepancies exist in the literature [as also stated in the manuscript, line 29] regarding the chromium local environment (oxidation state and chemical distribution when under soluble form) in DF, **<u>this study fails to bring new experimental and EXAFS-modelling insights</u>**.

… the **<u>presentation is incomplete and it is difficult, sometimes impossible, to understand what has been done</u>**. It is not clear from the paper, exactly what the authors have learned and what their objective is for the presentation. Consequently, the reviewer advises to reject the paper.

Author Comment: We thank the Reviewer for taking the time to thoroughly review our manuscript. We highlight three points in their general assessment for comment:

a. The Reviewer finds the subject worthy of study, and we are grateful for this assessment. Clearly, we are in agreement, particularly because (as Reviewer 1 notes) there exist many discrepancies regarding this topic in the literature.
b. They claim that our study fails to bring new experimental and EXAFS-modelling insights. **<u>We strongly dispute this claim!</u>** Indeed, there have been many attempts to resolve the information that we have successfully presented in our paper. There are

many discrepancies in the published literature, which arises from the fact that previous studies have focused on overly complex materials that are unsuitable to resolve the effects of numerous variables, whereas, our study present unambiguous evidence for the presence of Cr^{2+} in a model (simple) UO_2 system, corroborated by data from several different techniques. The majority of the comments of Reviewer 2 relate to their interpretation of our XAS data and they are easily rebutted.

c. The Reviewer finds the presentation of the work incomplete and difficult, but they do not give any details of which parts are incomplete or difficult, therefore, making it challenging for us to address this concern. The manuscript is written in the style of a communication and is intended to be brief. We note that Reviewer 3 stated that "the manuscript is written in a clear and concise style".

In the following, we offer responses to the specific comments of Reviewer 2, including details of changes to our manuscript. Where made, we feel that these changes strengthen our arguments.

Comment 1: Page 3 lines 88: When discussing the results reported in Ref. [23], the authors have mentioned ".......the data quality reported in Ref. 23 was not sufficient to obtain reliable evidence for the Cr speciation and local environment". The reviewer is not convinced, if this was the case, when the EXAFS oscillation spectrum and the corresponding FT-EXAFS spectrum in Fig. 2 (k-3 weighted data, up to k-range 13) in the manuscript are compared with those results reported in Ref. 23 (Fig. 3., k-2 weighted data, k-range up to 12).

Response 1: Although it was very much not our intention, we concede that our comment about the quality of the data in Ref 23 may be inappropriate, therefore, we have removed the offending text.

Comment 2: Page 8, line 252: When discussing the FT-EXAFS data and modeling using FEFF paths, the authors have stated "Previous work has experienced limited success in obtaining and analyzing XAS data (both XANES and EXAFS) on systems similar to this sintered sample. As such, this proposed model, generated using high flux, low-noise scanning EXAFS, provides insight to a possible structure for sintered Cr-doped UO2." Here, authors have cited three references: [Ref. 20, XANES data measured at ESRF using 300 micron beam spot, DF contains Cr2O3 precipitates, area probed 600 micron diameter disc], [Ref. 21, new experimental data is not reported by the authors, used the data published in Refs. 20 and 23, re-evaluated the XANES data, and unexpectedly found Cr(2+) species...] and [Ref. 23, data measured at SLS using 2 micron beam spot, DF contains Cr2O3 precipitates, ...].

It is found that in Ref. [23] the curve fitting result of multiple shell analysis is not reported and those authors have discussed what they believe. Therefore, the reviewer does not acknowledge the aforementioned statements. The reviewer is also aware that high flux, low-noise scanning EXAFS experiments are routinely performed at the ESRF and SLS.

Response 2: The Reviewer is concerned with the statement we make on line 252: "Previous work has experienced limited success in obtaining and analysing XAS data (both XANES and EXAFS) on systems similar to this sintered sample".

While we agree that data has been previously acquired on complex Cr-doped UO_2 samples (e.g. actual nuclear fuels), we are unconvinced that these data have been able to resolve the mechanism of Cr substitution in UO_2 because (a) the EXAFS fitted models have been limited by lack of supporting data (provided in our study by Raman spectroscopy and U M_4-edge XAS, plus new DFT insight from reference 21); and (b) the samples were, in their nature, overly complex and containing multiple Cr oxidation states as a function of sintering. This is why, in

our study, we claim to have resolved the structure, using a combination of techniques, using simple materials.

We have amended the text to clarify this, as follows:

Lines 254 to 260: While informative, without underpinning understanding from simplified systems like the calcined powder investigated in the present study, and the lack of corroborating analysis from other techniques, resolution of the coordination environment of Cr from previous XAS has proven challenging. As such, this proposed model, with supporting data from other techniques and fitting models grounded in DFT simulations, provides insight to a possible structure for sintered Cr-doped UO_2.

Comment 3: EPMA results [supplementary Fig. 3 (sintered pellets)] show that the prepared UO2 materials contain solute Cr and insoluble Cr in the form of submicron-sized second phase precipitate (SPP, Cr2O3 phase used in FEFF calculations). Regarding the calcined powder samples the authors have claimed on page 3 line 111, “All oxide powders were shown to contain the intended concentration of Cr when fully digested (in the range 300 to 2400 ppm)”.

It has been also stated in the manuscript (on page 6 line 173) “Given that the concentrations of Cr in the calcined material exceed the generally agreed solubility limit of Cr in UO2 reported to be in the range of 700 – 1200 ppm, it can be assumed that any excess Cr is present as positive interstitial defects”. The statements made are contradictory. However, the authors do not consider other possibilities, such as, local clustering of excess Cr (although unlikely, as mentioned on page 8 line 236 in the manuscript) and/or the presence of ultrafine chromium oxide particles in the calcined samples.

Response 3: These statements – that all the intended Cr was in the powders, and that some of it may reside in interstitial sites – are not contradictory… The as-prepared Cr-doped UO_2 calcined powders were dissolved in acid and the Cr concentration measured by ICP-MS. This technique does not discriminate *where* the Cr exists within the powders (i.e. lattice or interstitial sites). Later spectroscopic analysis we present suggests that not all of the Cr may be on U lattice positions. As such, both statements (that all the intended Cr was in the powders, and that some of it resides in interstitial sites), are complementary.

Comment 4: Cr K-edge XAS measurements are performed at the DLS using X-ray beam size of 400 micron. This means, apart from solute Cr in the analyzed materials, a large number of SPP located within the probed volume were also illuminated by the incident light and contributed to the measured Cr K-edge XAS spectra. This raises question on the reported FEFF modelling of measured EXAFS data for the calcined oxide powder, where any Cr contribution of precipitated Cr (Cr2O3 or any other possible form) has been neglected in the data analysis. How the authors know that all Cr are only in solid solution when the Cr-content have exceeded the solubility limit of Cr? Multiple ultrafine Cr-containing particles (in oxide phase or other form) could (and are likely to) be present in the materials. These points are not addressed in the paper.

Response 4: The Reviewer is concerned that there may be multiple species of Cr present within the calcined sample and that the size of the beam means that our data would sample all of these if present. We believe this not to be the case for the following reasons:

(1) The presence of Cr_2O_3 was not identified by any other technique in the calcined powders. It was, however, identified in the sintered samples (therefore highlighting that the techniques we used could adequately identify such phases).
(2) The results of the EXAFS modelling unambiguously suggest that the dominant speciation of Cr in the calcined material is fully described by the $1Cr+1O_v$ model. Whilst

this does not fully discount the presence of very small quantities of other Cr species, given the excellent statistics (e.g. goodness of fit) for the produced EXAFS model, and the multiple different unsuccessful attempts at fitting the data with different models, including those with Cr_2O_3 present, we are confident in the results we have presented here.

For clarity, on lines 139-142, we have made an addition to the text to state that we do not believe that the model arises from multiple phases:

Lines 139-142: Given the excellent statistics of the fit (R-factor and corroborating BVS), it is unlikely that any other Cr species exist in the calcined sample, with its bulk speciation being accurately described by the $1Cr^{2+} + 1O_v$ model.

Comment 5: XANES data: In this study, conclusions are ascribed to the effect of Cr oxidation state(s) to describe the chromium incorporation mechanism. However, no results of the detailed XANES analyses (first or second inflection point, first or second-derivative procedure, etc.) are provided in the manuscript. The assignment of oxidation number (+2) of all solute Cr is based only on the observed pre-edge feature in the XANES data. It is not a new result, previously reported in the literature. The Cr(2+) assignment, from the reassessment of the published XANES data reported in Ref. [21], is not fairly convincing [the spectral broadening due to the core-hole lifetime at the Cr K edge > 1 eV].

Response 5: The Reviewer wishes to see further analysis regarding the XANES data interpretation of Cr^{2+}. We are happy to add this to the supplementary information and have now provided edge positions for samples and standards for both the peak of the first derivative and 0.5 a.u. of the normalised edge step (Supplementary Data Table 2). However, it should be noted that due to the complexity of Cr K-edge XANES, the edge position is not diagnostic of oxidation state. As with all XANES data, other factors such as pre-edge features, charge transfer bands, and coordinating ligand type all influence both the shape and edge position of the XANES, however this is particularly pronounced in Cr K-edge XAS (for example, see Tromp et al., AIP Conference Proceedings, 882, 699 (2007)).

Supplementary Table 2. Cr K-edge XANES positions derived from the peak of the first derivative and 0.5 a.u. of the normalised edge step.

Category	Sample	Peak of the first derivative (eV; ± 0.1)	0.5 a.u. of the normalised edge step (eV; ± 0.1)
Standard	Cr(III)$_2$O$_3$	6006.0	6000.3
	Cr(II)UO$_4$	6003.2	6001.5
	Cr(0) Foil	5989.0	5994.0 – 5995.7
Calcined	300 ppm Cr	6005.5	6002.9
	600 ppm Cr	6003.2	6000.5
	1200 ppm Cr	6003.2	6000.8
	2400 ppm Cr	6003.7	6000.6
Sintered	300 ppm Cr	6000.4	5995.0
	600 ppm Cr	6000.2	5995.1
	1200 ppm Cr	6000.0	5995.4
	2400 ppm Cr	6000.1	5995.7

Comment 6: EXAFS data: The best fit of the FT-EXAFS data, measured from 2400 ppm Cr-containing calcined powder sample, was achieved by utilizing a 1Cr(2+)+1Ov model structure. This model is based on the assumption that most of the Cr atoms (say between 700 and 1200 ppm; as mentioned on page 6 line 175 in the manuscript) are dissolved in UO2, the material is precipitate free, and the authors think (or assume) that excess Cr is present as positive interstitial defects (line 175 in the manuscript) as predicted using DFT-based calculations [(Ref. 8 in the manuscript); to the best of this reviewer's recollection, there is no experimental result, reporting interstitial Cr content in DF, available in the open access literature]. However, the authors do not consider the contribution of those interstitial Cr atoms in their FT-EXAFS data analysis. Secondly, U(5+) has been identified in this sample [HERFD-XANES results of as-produced calcined material (Fig. 3b)], and the authors did not check a lattice model in its simplest form of 1Cr(3+)-U(4+) structure that can be directly related to compensate the charge imbalance when Cr(3+) is substituted for U(4+).

Response 6: The main point of the Reviewer is that our EXAFS model for calcined material does not take into account the presence of Cr interstitials. As noted in our response to Comment 4, the model that fit the EXAFS data the best did not involve Cr interstitials. This is unsurprising since we find no conclusive experimental evidence for the presence of Cr in interstitials, nor do we report it thus. We *suggest* (lines 171 – 178) that an increase in O_i could be related with Cr in interstitials, as previously hypothesised by reference 8, *or* it could be the result of oxidation of the sample during Raman analysis.

We checked numerous EXAFS models, including those that attempted to incorporate different Cr species within the calcined material structure. These produced qualitatively or quantitatively worse fits, chemically and/or physically unfeasible model structures, or lacked a significant means of justifying the presence of a given back-scatterer at a given interatomic distance. For these reasons, in addition to the compelling argument that the modelled CIF file used from the previously published work has been shown to be the most stable form of Cr in UO_2 and fit the data well, we have presented this as our final EXAFS fit in the manuscript.

An addition to the manuscript has been made to provide clarity surrounding the absence of Cr interstitials in the EXAFS fits:

Lines 178-179: Given that the EXAFS model did not require the presence of Cr interstitials to improve the fit, the latter explanation may be more reasonable.

Comment 7: For the EXAFS data of sintered pellet (2400 ppm Cr containing material), the best fit was achieved by utilizing the 1Cr(2+)+1Ov structure plus a 2Cr(2+)+2Ov structure for solute Cr, and a Cr3+ environment of Cr(3+)2O3 phase. In the manuscript (line 235) the authors have stated that EXAFS data analysis for this sample was challenging, determined the local coordination environment of chromium ions in the sample, and presented a complex structural model for solute Cr(2+) (65%) [35% is Cr(3+) of Cr2O3 phase] present in the sintered DF pellet in its final form. However, the authors do not discuss or report any structural analyses of other possible structure candidates [such as, 2Cr(3+)+1Ov for solute Cr, a mixed 2Cr(3+)+1Ov and Cr(3+)2O3 model, ..] that have been also suggested in the literature.

Response 7: See also response to Comment 6. During the processing of our data, we trialled a large variety of fits and chose the best and final fit for inclusion in the manuscript. Noting that this is a communication style paper, it precludes the inclusion of every single unsuccessful fit. However, we appreciate that observation of unsuccessful fits can sometimes lend credibility to the final chosen fit. Therefore, we have added information about a selection of unsuccessful fits to the Supplementary Data including a simple one O shell fit for direct comparison with

previous work by Mieszczynski *et al.* (2014) and fits based upon some of the alternate models provided by Sun *et al.* (2020) – see Supplementary Data Fig. 6 and Supplementary Data Table 1. Also, lines 233 – 236 of the manuscript have been altered to direct the reader to this information.

Lines 234-237: ‘Several unsuccessful models were explored (a selection of which are shown in Supplementary Data Fig. 6 and Supplementary Data Table 1)), including simple fits that only contain oxygen backscatterers, and fits based upon alternate models from the work of Sun *et al.*[21]’

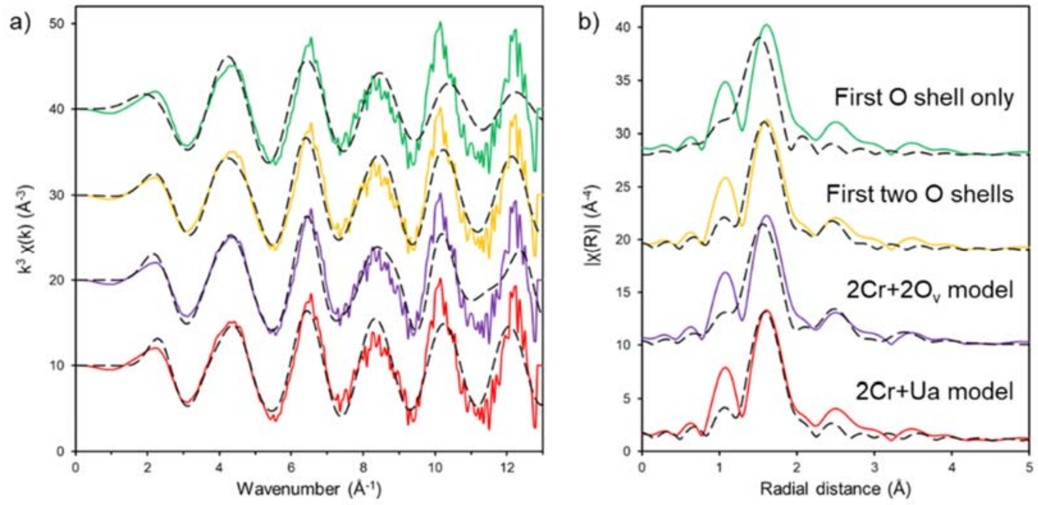

Supplementary Figure 6. Comparison of a selection of different fitting models for the Cr-doped UO_2 calcined powder (2400 ppm) trialled during EXAFS modelling. a k^3-weighted Cr K-edge spectra fit between 3-11.5 and; **b** the associated Fourier transform fit between 1.35 - 3 for the ‘First O shell only’ and ‘First two O shells’, and ‘2Cr+2O_v model’ and ‘2Cr+Ua model’ were fit between 1.35 – 3.9. **‘**First O shell only’ and ‘First two O shells’ utilised the 1Cr + 1O_v CIF file used in the main manuscript. ‘First O shell only’ fit acts as a direct comparison with the EXAFS fitting model employed in Mieszczynski *et al.* (2014). ‘2Cr+2O_v’ and ‘2Cr+Ua’ models are derived from other CIF files from the work by Sun *et al.* (2020). See Supplementary Table 1 for fitting data.

Supplementary Table 1. EXAFS fitting parameters for the different fitting models shown in Supplementary Figure 5 for Cr-doped UO_2 calcined powder (2400 ppm). 'First O shell only' and 'First two O shells' utilised the 1Cr + $1O_v$ CIF file used in the main manuscript. 'First O shell only' fit acts as a direct comparison with the EXAFS fitting model employed in Mieszczynski *et al.* (2014). '2Cr+$2O_v$' and '2Cr+Ua' models are derived from other CIF files from the work by Sun *et al.* (2020). (S_0^2) is the amplitude reduction factor, (ΔE_0) the shift from Cr K-edge position (5989 eV), (N) the degeneracy, (R) (Å) the fitted bond length, and (σ^2) the Debye-Waller factor.

	First O shell only	First two O shells	2Cr+$2O_v$ model	2Cr+Ua model
S_0^2	0.90	0.90	0.90	0.90
ΔE_o	-7.7(43)	-1.8(22)	-3.1(24)	-0.1(35)
N (Cr-O1)	6	4	5	4
R(Cr-O1)	1.97(2)	1.99(1)	1.99(1)	2.00(2)
σ^2 (O1)	0.005(2)	0.001(1)	0.003(1)	0.001(1)
N (Cr-O2)	-	3	2	8
R(Cr-O2)	-	2.96(4)	-0.001(3)	0.027(25)
σ^2 (O2)	-	0.005(6)	2.93(3)	3.92(17)
N (Cr-U1)	-	-	4	4
R(Cr-U1)	-	-	3.54(6)	2.74(18)
σ^2 (U1)	-	-	0.017(9)	0.056(50)
N (Cr-U2)	-	-	2	-
R(Cr-U2)	-	-	3.83(11)	-
σ^2 (U2)	-	-	0.017(15)	-
N (Cr-Cr1)	-	-	-	1
R(Cr-Cr1)	-	-	-	2.51(7)
σ^2 (Cr1)	-	-	-	0.016(12)
N (O1-U1 MS)	-	-	-	16
R (O1-U1 MS)	-	-	-	3.34
σ^2 (O1-U1 MS))	-	-	-	0.057
R-factor	0.145	0.034	0.041	0.039
Bond valence sum (total)	3.172	2.070	2.574	1.925

Comment 8: EXAFS results and structural parameters reported in Table 1: The local atomic environment around Cr atoms, in calcined DF powder and sintered DF pellet, is examined by analyzing the EXAFS part of the XAS data. A multi-shell fitting approach has been employed for data fitting: four coordination shells (Cr-O1, Cr-O2, Cr-U1 and Cr-U2) for the calcined powder sample (containing 2400 ppm Cr); and five coordination shells (Cr-O1, Cr-O2, Cr-U1, Cr-U2 and Cr-U3) for the sintered sample (containing 2400 ppm Cr). All fits used the site-selective three (?) free parameters (N, R, and sigma) contained in each single-scattering FEFF path included in the data analysis. Knowing the fact that the said parameters are strongly correlated in the EXAFS equation.

Response 8: This comment relates the EXAFS fitting procedure for our data and we have broken it down into sections for ease of response. At a high level, the Reviewer questions our

application of quite standard methodology, widely applied. We feel that this is wholly justified and our explanation for this is given, point-by-point below. Some of these questions are easily addressed by including additional information about the fits in the Supplementary Data.

Comment 8 (a) the EXAFS-derived numerical values of structural parameters reported in Table 1 are questionable since the total number of free parameters are 12 and 15 (+ additional FEFF paths taken for the undissolved Cr2O3 phase), respectively, for the two experimental data-set analyzed;

Response 8 (a): The Reviewer questions the number of parameters used in our fit and which parameters were allowed to refine. Our approach is justified two-fold: (1) the degeneracies used for each shell were fixed in accordance with the predicted structure from the CIF file to ensure that the Nyquist criterion was satisfied and that the resulting EXAFS model had physically and chemically meaningful foundation; and (2) the parameters that were floated were R (change in interatomic distance), E_0 (change in Fermi level energy), and σ^2 (Debye-Waller factor). This methodology is commonly used and has been published widely.

Comment 8 (b) reason for the assumption of an overall delta-E0 (-0.80 eV for the calcined powder and -6.0 eV for the sintered sample) to be the same for both Cr-O1(or O2) and Cr-U1(or U2 and U3) scatterings paths is not clear, and why this parameter was constrained during the final refinements;

Response 8 (b): If this assumption were not made, i.e. a separate E_0 is given to O and U backscatterers, the model could lose physical and chemical meaning, in addition to violating the Nyquist criterion. There may be some argument to suggest a separate E_0 should be used for different oxidation states (i.e. in the sintered sample) but this is not a requirement and merely an option to utilise and in an attempt to improve a poorly fitting model. This method is standard practise for EXAFS fitting as means of constraining the number of parameters used and maintaining physical and chemical meaning of the produced model, as detailed in numerous publications including 'EXAFS for Everyone' by Scott Calvin, and 'Quantitative EXAFS Analysis' by Bruce Ravel.

Comment 8 (c) most importantly, how the authors know that multiple-scattering (MS) effects should be excluded [(MS) FEFF paths] in their data analysis. The authors have neglected the MS contribution in their data analysis, and do not provide any justification for the same in their manuscript;

Response 8 (c): Contrary to the Reviewer's assertion, multiple scattering (MS) paths were considered during the fitting process. We have included a fit with the inclusion of a MS path in the Supplementary Data (Fig. 6 and Table 1). However, there were few multiple scattering paths with significant enough rank (≤ 10 %) to merit their addition to the fit given the number of high rank single scatterer paths that dominated the XAS spectra. It is not unusual for EXAFS models to only be constructed using single scattering paths as these are often paths that dominate the spectra (as is the case here) and, as with the other points raised, this is a standard practise within the EXAFS modelling community.

Comment 8 (d) although the complex structure of the FT-EXAFS spectra (Fig. 2b and Fig. 2e) indicates a structurally complicated Cr local environment in the samples, a shell-by-shell fit of Fourier-filtered data was not attempted by the authors;

Response 8 (d): Contrary to Reviewer's assertion, shell-by-shell fitting was indeed attempted. We have included additional information, through the presentation of simple O-only fits in the Supplementary Data (Fig. 6 and Table 1) to highlight this. This method was ultimately unsuccessful due to the reasoning provided by the Reviewer; the structural model was

complicated. It is not unusual to be unable to fit EXAFS data in a shell-by-shell model because this approach assumes that only one type of backscatterer influences the spectral intensity of one FT peak (shell). This assumption, outside of simple well-constrained standard systems, is often flawed as other backscatterers (particularly heavier elements such as U) have a strong influence over the overall EXAFS spectrum and so a more holistic approach to producing a model is required. This is the case for the spectra here (and is indeed the case for many other EXAFS models produced throughout the literature) and, as such, a shell-by-shell approach was unsuccessful and so these attempts were not provided in the manuscript.

Comment 8 (e) assuming that the strong peak near 1.5 A in Fig. 2b (calcined sample) and 2e (sintered sample) is due to the first coordination Cr-O shell, the authors did not assign the visible FT peak near 1 A in the data (Fig. 2b) that is, however, absent in Fig. 2e;

Response 8 (e): The peak at 1 Å has not been assigned as this is a feature of the background function that is applied in pre-processing of the data before fitting. It is simply not physically meaningful – if fitted, it would correspond to an interatomic distance that would place a hypothetical backscatterer too close to the central absorbing Cr atom (~1.4 Å; shortest Cr-O bond distance reported are ~1.7 Å).

Comment 8 (f) According to the fit results of both samples in Table 1, the coordination number of 7 has been determined and the Cr-O bond length distribution is bimodal in the immediate neighborhood of the dopant ions. However, for the second coordination Cr-U shell [equivalent U-U, N=12, crystalline UO2], the fit results of DF materials show highly undercoordinated chromium sites with only 6 U atoms [N=6 [(Cr-U1)+(Cr-U2)+(Cr-U3)]] instead of 12 U atoms, and the situation corresponds to a trimodal distribution of bond lengths. The origin of these anomalies of the atomic coordination parameters are not discussed in the paper.

Response 8 (f): On the contrary, we clearly describe the degeneracies of oxygen and uranium backscatterer, please see lines 124-142.

Reviewers' comments:

Reviewer #1 (Remarks to the Author):

Authors' answers are satisfactory with regards to the current reviewer's comments. However, any comments from other reviewers should be answered before the acceptance of the manuscript in its current condition.

Reviewer #2 (Remarks to the Author):

Editorial note: please see attachment.

Reviewer #3 (Remarks to the Author):

The authors have taken in consideration all the comments made by the reviewers, leading to improve a manuscript that was already of high interest inits first version.
As such, I recommand it for publication without any further modification.

Received the easily rebutted response letter and the revised manuscript. In this case, the following (selective only) comments are given.

The authors have mentioned that they have prepared a simple (model) material (up to 2400 ppm Cr containing calcined DF powder). Here, Cr-content is expected to exceed the solubility limit of Cr. They have also mentioned; (Response 3) …. *spectroscopic analysis we present suggests that not all of the Cr may be on U lattice positions.* For these situations, they have analyzed the EXAFS data, to show divalent Cr(2+) solution in UO2 system, considering only Cr(2+)+Ov model structures. Such approximation has become evidently inappropriate.

In the revised manuscript the authors have added the supplementary Figure 6 and supplementary Table 1 for Cr-doped UO2 calcined powder (2400 ppm) sample, and stated (line 632): *"First O shell only' ….. utilised the 1Cr + 1Ov CIF file used in the main manuscript. 'First O shell only' fit acts as a direct comparison with the EXAFS fitting model employed in Ref. [23, Mieszczynski et al. (2014)]".* Not at all. In Ref. 23, one can find that those authors did not use 1Cr + 1Ov model in FEFF9 calculation for FT-EXAFS data analysis, and considered the given results [1Cr(3+)-U(4+) structure that can be associated with U(5+)] from Refs. 15 and 16 (in the revised manuscript). (a) Therefore, the fitted result shown in the supplementary Figure 6 is not directly comparable with that was reported in Ref. 23, AND this second point in comment 6 is not addressed in the revised manuscript. (b) Related to this *First O shell only* fitting result, in the supplementary new Table 1, the reported N value for the Cr-O1 shell is 6 (it remains unclear if this value is a constrained value or a value obtained from the best-fit). For 'First O shell only' analysis using the 1Cr + 1Ov model, the N value is expected to be 7 [if not floated]. And, in the response letter (response 8a) the authors have mentioned that: *the parameters that were floated were R, E0 and σ2*]. Whereas, the authors have mentioned in the revised manuscript (line 409) that: …….. *the following parameters allowed to refine; N, R, E0 and σ2*. Not clear that has been done.

For sintered DF sample, the authors have also used the same model structure and methodology. In the revised manuscript (lines 257-259) the authors have stated: "*As such, this proposed model with supporting data from other techniques* (i.e., Raman spectroscopy and U M4-edge XAS) *and fitting models grounded in DFT simulations* [i.e., Ref. 21, Sun et al., Commun. Mater. 1, (2020) 1], *provides insight to a possible structure for sintered Cr-doped UO2"*. (a) The results of Raman spectroscopy study (with Principal Component Analysis) for sintered DF materials are already available in the open literature [published last year, Ref. [11], Milena-Pérez et al., J. Nucl. Mater. 543 (2021) 152581], where those authors have presented results related to the Cr solubility limit (about 750 ppm), and structural Ov-defects creation due to the entry of Cr(3+) in the UO2 matrix. This recent Raman results suggest that a 2Cr(3+)+1Ov model for solute Cr, and a mixed 2Cr(3+)+1Ov plus Cr(3+) environment of Cr(3+)2O3 model to be tested in FT-EXAFS analysis for the sintered sample. These points are not addressed in the revised manuscript (comment and response 7). (b) In addition to the new DFT insight from reference 21 (Response 2); it may be noted that DFT insight is also available in the open literature [Ref. 16, Middleburgh et al., J. Nucl. Mater. 420 (2012) 258] where those authors have concluded: "DFT calculations have shown that chromium will maintain its 3+ charge within UO2, charge compensated by an adjacent uranium ion in a 5+ charge state". This theoretical result can be corroborated by associating the experimental U M4-edge XAS result presented in Figure 3 of this manuscript.

In summary, information required to place the present work in the context of previous work is missing. All raised points are not satisfactorily addressed in the revised manuscript. In addition, the reviewer does not find convincing arguments in the response letter (including those in response 4, 6, 7 and 8).

Dear Editor,

Reviewer 2 makes one general and two specific comments that we address in blue text below. We make only one very minor change to the manuscript in response.

General comment: …they have analyzed the EXAFS data, to show divalent Cr(2+) solution in UO_2 system, considering only Cr(2+)+Ov model structures. Such approximation has become evidently inappropriate.

General comment response: We believe that our manuscript presents strong evidence for the presence of Cr^{2+} when substituted into the UO_2 matrix. The Reviewer disagrees, and we feel that this stems from their misunderstanding / misinterpretation of EXAFS modelling and the model fits that we present in our manuscript. This is exemplified by their **Comment 1a** in the current rebuttal, in which they argue we have not correctly represented the model in Reference 23 in our manuscript, whereas, we used the *exact same model*, albeit with a different name (see response to **Comment 1a** below).

We accept that one part of our model explanation required amendment for clarity and are grateful to the Reviewer for highlighting this in the their most recent review (see response to **Comment 1b** in the current rebuttal). This change is highlighted in the revised manuscript in red text.

We welcome the challenge of Reviewer 2 in regard to Cr oxidation state, not least because it serves to highlight that significant ambiguity persists in the literature regarding the oxidation state of Cr in UO_2, with various authors postulating Cr^{1+}, Cr^{2+} and Cr^{3+} (see response to **Comment 2a and b** in the current rebuttal). The other Reviewers (1 and 3) were happy to accept our arguments for the presence of Cr^{2+} as being based on multiple, well-reasoned lines of evidence.

To provide additional evidence for our interpretation of Cr substituted in the structure as 2+, we attach to this submission another of our manuscripts that is currently under review (in ACS Applied Materials and Interfaces). In this work, we evidence the presence of Cr^{2+} through observation of galvanic coupling between Cr^{2+} with U^{6+} in the corrosion of Cr-doped UO_2. The galvanic couple between Cr^{3+} and U^{6+} is thermodynamically impossible (a non-spontaneous reaction) and, therefore, there must be Cr^{2+} present in Cr-doped UO_2 to explain our observations. We appreciate that Reviewer 2 did not have access to this additional manuscript at the time of their review(s), which may have helped to relieve their apparent doubts about our conclusion of Cr^{2+}.

Comment 1a: In the revised manuscript the authors have added the supplementary Figure 6 and supplementary Table 1 for Cr-doped UO2 calcined powder (2400 ppm) sample, and stated (line 632): "First O shell only' ….. utilised the 1Cr + 1Ov CIF file used in the main manuscript. 'First O shell only' fit acts as a direct comparison with the EXAFS fitting model employed in Ref. [23, Mieszczynski et al. (2014)]". **Not at all**. In Ref. 23, one can find that those authors did not use 1Cr + 1Ov model in FEFF9 calculation for FT-EXAFS data analysis, and considered the given results [1Cr(3+)-U(4+) structure that can be associated with U(5+)] from Refs. 15 and 16 (in the revised manuscript). Therefore, the fitted result shown in the supplementary Figure 6 is not directly comparable with that was reported in Ref. 23 AND this second point in comment 6 is not addressed in the revised manuscript.

Response 1a: The key point of relevance is that our data and those of reference 23, (which are used by those authors to conclude that Cr is present as Cr^{3+}) are completely different –

see Figure (i) below and note the different waveforms. Irrespective of how one fits our data to the EXAFS model derived from Reference 23, it will never fit well. We show this by including all possible fits, including the 1Cr(3+)-U(4+) fit described in Reference 23, in Supplementary Figure 6 and Supplementary Table 1. We call this model a “First O shell fitting result” since it describes the Cr-O path used in the 1Cr(3+)-U(4+) fit, as per the nomenclature of the authors of reference 23 (“the first-shell signal”) shown in the screenshot of the text below.

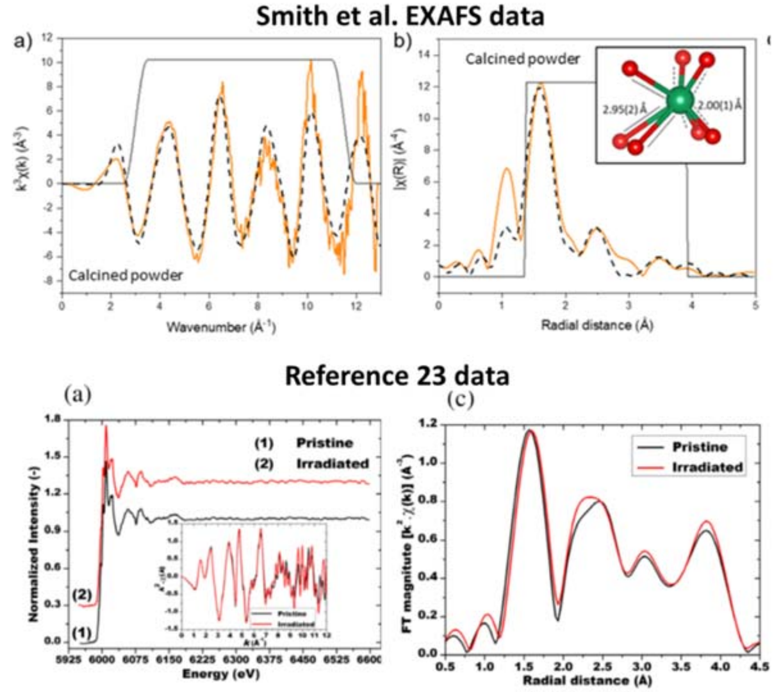

Figure (i) Comparison of Cr K-edge EXAFS data and model fits for **TOP** Smith et al., data shown in orange, modelled fit in dashed black lines; and **BOTTOM** Reference 23, only data are shown. The differences between the EXAFS data are manifest by the different waveforms (right and inset) and the corresponding Fourier Transforms (left).

> With respect to an easy derivation of Cr structural parameters, we also analyzed only the first-shell signal of Cr K-edge EXAFS data. After extracting the Cr–oxygen shell component from radial distribution functions (see figure 3(*c*)), the back-transformed spectra were best-fitted using R, CN, and σ as all free parameters, which is a more realistic approach. The fitted curves represent the experimental data, which are depicted well in figure 4. The quantitative fittings of the nearest oxygen neighbor in the pristine and irradiated UO_2 samples give the average bond lengths and coordination numbers around the Cr atoms; those results are summarized in table 1. From this

We feel that we clearly answered Comment 6 of the Reviewer in our previous response but don’t appear to have arrived at a common understanding. We will of course elaborate on our previous rebuttal if the Editor wishes, however, it is our contention, as demonstrated above, that this point is of nugatory significance in terms of the conclusions of the paper.

Comment 1b: Related to this First O shell only fitting result, in the supplementary new Table 1, the reported N value for the Cr-O1 shell is 6 (it remains unclear if this value is a constrained value or a value obtained from the best-fit). For ‘First O shell only’ analysis using the 1Cr + 1Ov model, the N value is expected to be 7 [if not floated]. And, in the response letter

(response 8a) the authors have mentioned that: the parameters that were floated were R, E0 and σ2]. Whereas, the authors have mentioned in the revised manuscript (line 409) that: …….. the following parameters allowed to refine; N, R, E0 and σ2. Not clear that has been done.

Response 1b: When one is performing an EXAFS fit, certain values are allowed to refine, while others are fixed. In the First O shell only fitting result, we fixed the value of N as 6 to exactly replicate the so-called 1Cr(3+)-U(4+) model of Reference 23. For the 1Cr+1Ov CIF file, the total coordination was fixed at 7.

We agree that information regarding which parameters were fixed or refined was unclear. We have amended the text in the methods section (lines 409 – 410 and 414 – 415) to resolve this.

Comment 2: We understand this comment to relate to appropriate citation of, and comparison of our data with, other literature. We have broken down our response for ease of reply:

Comment 2(a) For sintered DF sample, the authors have also used the same model structure and methodology. In the revised manuscript (lines 257-259) the authors have stated: "*As such, this proposed model with supporting data fro other techniques* (i.e., Raman spectroscopy and U M4-edge XAS) *and fitting models grounded in DFT simulations* [i.e., Ref. 21, Sun et al., Commun. Mater. 1, (2020) 1], *provides insight to a possible structure for sintered Crdoped UO2"*. (a) The results of Raman spectroscopy study (with Principal Component Analysis) for sintered DF materials are already available in the open literature [published last year, Ref. [11], Milena-Pérez et al., J. Nucl. Mater. 543 (2021) 152581], where those authors have presented results related to the Cr solubility limit (about 750 ppm), and structural Ov-defects creation due to the entry of Cr(3+) in the UO2 matrix. This recent Raman results suggest that a 2Cr(3+)+1Ov model for solute Cr, and a mixed 2Cr(3+)+1Ov plus Cr(3+) environment of Cr(3+)2O3 model to be tested in FT-EXAFS analysis for the sintered sample. These points are not addressed in the revised manuscript (comment and response 7).

Response 2a: Milena-Pérez et al., present a study of sintered Cr-UO_2, in which they make **no measurement of Cr oxidation state**. It is, therefore, impossible to guess correctly which EXAFS models might be derived from this work. It would be futile to base our EXAFS models upon guess work, hence we do not consider it in our manuscript.

Comment 2(b) In addition to the new DFT insight from reference 21 (Response 2); it may be noted that DFT insight is also available in the open literature [Ref. 16, Middleburgh et al., J. Nucl. Mater. 420 (2012) 258] where those authors have concluded: "DFT calculations have shown that chromium will maintain its 3+ charge within UO2, charge compensated by an adjacent uranium ion in a 5+ charge state". This theoretical result can be corroborated by associating the experimental U M4-edge XAS result presented in Figure 3 of this manuscript.

Response 2b: We agree that some published DFT studies do propose Cr^{3+} as the oxidation state of Cr in UO_2. There are also DFT studies that propose it to be Cr^{2+} (reference 21) and even also those that postulate Cr^{1+} (reference 8). On the contrary to the Reviewer's suggestion, the U M_4-edge data presented in our manuscript disprove the hypothesis of reference 16 (who assert Cr^{3+}), as we show that when Cr^{3+} is present, in sintered samples, there is no U^{5+}. This serves to highlight the valuable addition of our experimental data to the literature, to provide additional experimental insight to resolve the uncertainty surrounding the oxidation state of Cr in UO_2.
